# Repeated inoculation with rumen fluid accelerates the rumen bacterial transition with no benefit on production performance in postpartum Holstein dairy cows

**DOI:** 10.1186/s40104-023-00963-9

**Published:** 2024-02-04

**Authors:** Fanlin Kong, Feiran Wang, Yijia Zhang, Shuo Wang, Wei Wang, Shengli Li

**Affiliations:** 1 Beijing Engineering Technology Research Center of Raw Milk Quality and Safety Control, State Key Laboratory of Animal Nutrition, Department of Animal Nutrition and Feed Science, College of Animal Science and Technology, China Agricultural University, Beijing, 100193 PR China; 2 College of Veterinary Medicine, Nanjing Agricultural University, Nanjing, Jiangsu 210095 PR China

**Keywords:** Dairy cow, Lipidomics, Liver, Rumen microbiota transplantation, Transition period

## Abstract

**Background:**

The dairy cow’s postpartum period is characterized by dramatic physiological changes, therefore imposing severe challenges on the animal for maintaining health and milk output. The dynamics of the ruminal microbiota are also tremendous and may play a crucial role in lactation launch. We aim to investigate the potential benefits of early microbial intervention by fresh rumen microbiota transplantation (RMT) and sterile RMT in postpartum dairy cows. Twelve fistulated peak-lactation dairy cows were selected to be the donors for rumen fluid collection. Thirty postpartum cows were divided into 3 groups as the transplantation receptors respectively receiving 10 L fresh rumen fluid (FR), 10 L sterile rumen fluid (SR), or 10 L saline (CON) during 3 d after calving.

**Results:**

Production performance, plasma indices, plasma lipidome, ruminal microbiome, and liver transcriptome were recorded. After fresh and sterile RMT, we found that the molar proportion of propionic acid was increased on d 7 in the FR and SR groups and the bacterial composition was also significantly changed when compared with the CON group. A similarity analysis showed that the similarities between the CON group and FR or SR group on d 7 were 48.40% or 47.85%, whereas the similarities between microbiota on d 7 and 21 in the FR and SR groups were 68.34% or 66.85%. Dry matter intake and feed efficiency were not affected by treatments. Plasma β-hydroxybutyrate concentration in the FR group was decreased and significantly different lipids mainly included phosphatidylcholine and lysophosphatidylcholine containing polyunsaturated fatty acids. Hepatic transcriptomics analysis indicated acute-phase response pathways were upregulated in the SR group.

**Conclusions:**

Our study suggests that RMT can shorten the transition process of the ruminal microbiota of postpartum dairy cows with no benefit on dry matter intake or feed efficiency. Inoculation with rumen fluid may not be a useful approach to promote the recovery of postpartum dairy cows.

**Graphical Abstract:**

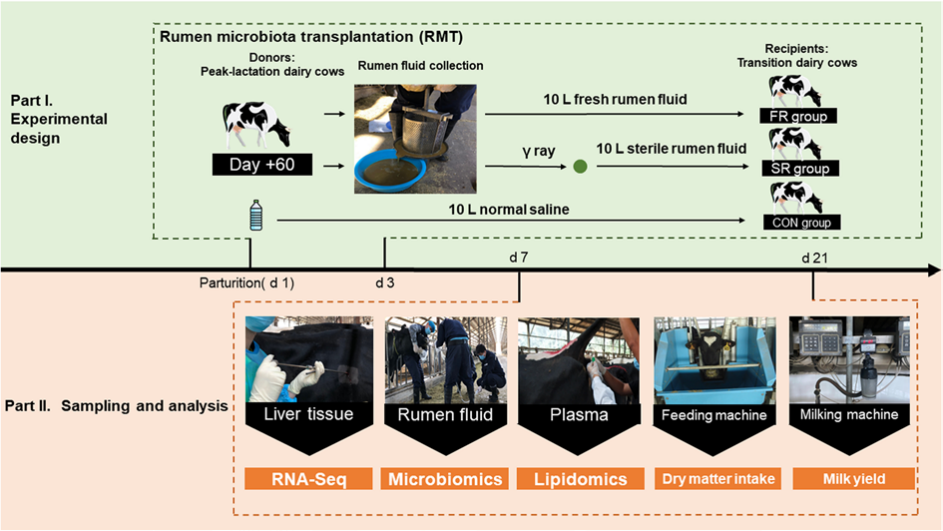

**Supplementary Information:**

The online version contains supplementary material available at 10.1186/s40104-023-00963-9.

## Background

The lifespan of dairy cows is about 20 years under natural conditions [1]. However, the average lifespan is about 3 to 4 years in dairy farms with intensive systems, which is still shorter than the economically optimal productive lifespan (5 years) [2]. The transition period is defined as the period from 3 weeks before to 3 weeks after parturition, and this period is considered a “window phase” to adapt to the metabolic changes from no-lactating to lactating and diet changes from lipogenic diet to glycogenic diet [3]. It is self-evidence that the inability of dairy cows to successfully adapt to lactation may contribute to unwilling elimination characterized by glucolipid metabolism disorder followed by low yield and reproductive failure [4]. Although animal nutritionists during the last decade have devoted themselves to revealing the mechanism of metabolic changes and improving the adaptation of dairy cows in the transition period, there is still a disproportionate amount of health care and culling occurs early following parturition [5, 6].

The ecosystem formed by abundant microbes in the rumen represents a classic model of host-microbiome interaction. Sufficient evidence has demonstrated that the rumen microbiome is linked to host metabolism [7, 8], immunologic function [7], inflammatory response [9], and production efficiency [10, 11]. Due to pregnancy stress and diet change, the microbiota in the rumen detected by high through sequencing found that fibrinolytic microbes hold a dominant position in the prepartum rumen ecosystem, while transition occurred during 2–3 weeks in the postpartum period and showed the overrepresentation of proteolytic, amylolytic and lactate-producer microbes [10, 12, 13]. The correlation analysis between these microbes and host parameters such as milk yield, and dry matter intake (DMI) indicated a close connection of the microbiome with dairy cows [10, 14]. Hence, we speculated that the maturation process of microbiota after parturition may influence host metabolism and immune response. However, only the correlation relationship rather than the causal relationship was provided in these studies between rumen microbiome and host performance. Scant information is known on whether promoting the transition progress of rumen microbiota contributes to postpartum recovery.

Fecal microbiota transplantation (FMT) is an effective method to manipulate the gut microbiota and, has been established to prove the role of microbiota in the development of diseases by delivering stool microbiota from a healthy individual to a patient, or in turn [15]. For ruminants, rumen microbiota transplantation (RMT) has shown potential value in treating diseases. DePeters and George [16] reviewed that 10–16 L rumen fluid transported to dairy cows could treat the indigestion of ruminants. The 10 L rumen fluid transplantation also improved feed intake and milk yield of cows after surgical correction of left-sided displacement of abomasum [17]. Moreover, a series of studies repeatedly inoculated fresh rumen fluid from adult ruminants for young ruminants to modulate the microbial development or weaning process, and results indicated the fresh rumen fluid was effective in accelerating the development process of young ruminants, while the sterilized rumen fluid had no effects [18–20]. Considering the similar microbial development process between the postpartum period and the young stage. Previous studies proved the benefits of RMT, but information regarding the application of RMT in postpartum dairy cows has not been well described.

We hypothesized that RMT could shorten the transition process of rumen microbiota after parturition and further improve metabolic adaption and recovery of DMI. To test this hypothesis, the peak-lactating dairy cows were used to provide mature and stable rumen microbiota for fresh dairy cows and we monitored the longitudinal changes of bacterial composition (16S rRNA) after RMT to certify the feasibility of RMT. Furthermore, the effects of the ruminal bacterial community on postpartum recovery by detecting production performance, plasma lipidome, and liver transcriptome (targeted lipidomics and RNA-seq) were further tested. We aimed to investigate the possibility of RMT and the role of rumen microbiota in postpartum recovery. Except for fresh rumen fluid in the RMT technology, the sterilized rumen fluid was also used as a transplant to distinguish the effects of live bacteria and metabolites.

## Methods

### Animals, housing, and diets

All experimental procedures were approved by the Institutional Experimental Animal Care and Use Committee of the Ministry of Agriculture and Rural Affairs of China and the Animal Care and Use Committee at China Agricultural University (issue number: AW01103202-1-32). The study was carried out on the experiment farm of the China Agricultural University (Beijing, China) with a herd size of 2,000 cows. Experimental dairy cows were removed from the dry-cow barn to the experimental barn at the prepartum to adjust to experimental conditions. Cows were fed twice daily (0700 and 1400 h) at 105% of expected intake and milked thrice daily (0600, 1400, and 1900 h). All cows received a high-grain diet after parturition and were fed as a total mixed ration (TMR) ad libitum. Diet was formulated to meet cow’s requirements according to NRC [21], and their ingredients and chemical compositions are presented in Additional file 1. All experimental cows had not received antibiotics for the past 6 months and formal experimental period (from calving to d 21).

### Experimental design and treatments

Twelve cannulated multiparous Holstein cows during the peak-lactation period (parity: 2.1 ± 0.4; days in milk: 59.67 ± 0.47 d; age: 4.1 ± 0.3 years) were used as donors of rumen fluid. These cows were selected before calving to keep similar predictive calving day and 305 d milk production and installed fistula during the dry period. After calving, all cannulated cows received standard operating procedures in this commercial farm until we collected rumen fluid. Thirty multiparous Holstein cows (parity: 2.5 ± 0.5; body condition score: 3.38 ± 0.24) were used in this completely randomized design and were assigned to 3 experimental groups as follows: FR group, SR group, and CON group. The cows in the FR group were dosing 10 L fresh rumen fluid daily from 1 to 3 d after parturition. The cows in the SR group were dosing 10 L γ-ray sterilized rumen fluid daily. Cows in the CON group received 10 L saline daily. The donors and receptors were fed the same diet. The RMT process is shown in Additional file 2. Briefly, a total of 12 people worked together and we collected the rumen content by hand from all donors before morning feeding and packed it via four layers of gauze. Then, a self-made steel juice extractor was used to extrude fresh rumen fluid. About 30 L of rumen fluid from the individual donor was obtained and pooled together (360 L). It means that the original rumen fluid for RMT in the FR group and SR group was the same. After mixing, we treated half of the fresh rumen fluid with γ-ray (25 kGy, 10 h, 37 °C, Beijing HYSF Technology Co., Ltd., Beijing, China). The microbial count was changed from more than 2 × 10^7^ to 10 CFU/mL by enumerating rumen bacteria according to the previous method [22]. Finally, a total of 10 L per cow of sterilized rumen fluid was transported into the rumen of dairy cows in the SR group by a manual fluid pump equipped with a plastic pipe. Half of the fresh rumen fluid and saline were transported immediately within 15 min to minimize the negative effects of oxygen exposure on the rumen microbiota. The collection of rumen fluid from donors, γ-ray treatment, and RMT was conducted every day during the first 3 d after calving.

### Sample collection and analysis

Samples of TMR were collected weekly and stored at −20 °C to further analyze nutrient composition according to Kong et al. [23] and the results were shown in Additional file 1. Milk production and DMI were recorded daily. Individual data were obtained by 9J-JI automated milking machine (2 columns × 48 seats, BouMatic Company, Madison, WI, USA) and automatic weighing feeding machine (RIC roughage intake control system, Hokofarm Group, Emmeloord, The Netherlands). The feed efficiency was calculated by dividing milk production by DMI.

Blood samples were taken on d 7 and 21 at 0500 h (before morning feeding). Blood was drawn from the coccygeal vein into a vacuum tube containing ethylene diamine tetraacetic acid (Beijing Laibotairui Technology Development Co., Ltd., Beijing, China) and, centrifuged (4,000 × *g*, 15 min, 4 °C) to obtain plasma and stored at −20 °C to further determine the biochemical indices. The subsample of plasma was stored at −80 °C to determine the lipidome. Concentrations of β-hydroxybutyric acid (BHBA), nonesterified fatty acid (NEFA), glucose (GLU), triglyceride (TG), total cholesterol (TC), glutathion peroxidase (GSH-Px), superoxide dismutase (SOD), malondialdehyde (MDA), total antioxidant capacity (T-AOC) were determined by colorimetric method and using fully automatic biochemical analyzer (GF-D200, Gaomi Analytical Instrument Co., Ltd., Gaomi, China) combined with commercial kits (Nanjing Jiancheng Bioengineering Institute, Nanjing, China). Concentrations of cortisol (Cort), immunoglobulin A (IgA), immunoglobulin G (IgG), immunoglobulin M (IgM), D-lactic acid (D-LA), diamine oxidase (DAO), lipopolysaccharide (LPS), serum amyloid A (SAA), tumor necrosis factor-α (TNF-α) were determined by ELISA kits (Beijing Laibotairui Technology Development Co., Ltd., Beijing, China). The quantitative insulin sensitivity check index (RQUICKI) was calculated according to Holtenius and Holtenius [24].

For the lipidome analysis, a 100 µL sample was transferred to an EP tube and then added with 480 µL extract solution (MTBE:methanol = 5:1, ANPEL laboratory technologies Inc., Shanghai, China). After vortex for 30 s, samples were sonicated for 10 min in an ice-water bath, then incubated at −40 °C for 1 h, and centrifuged at 3,000 r/min for 15 min at 4 °C (Heraeus Fresco17, Thermo Fisher Scientific, Waltham, MA, USA). A total of 300 µL supernatant was transferred to a fresh tube and dried in a vacuum concentrator at 37 °C. The dried samples were reconstituted in 100 µL of 50% methanol in dichloromethane (Merck KGaA, Darmstadt, Germany). The constitution was then centrifuged at 12,000 r/min for 15 min at 4 °C (Heraeus Fresco17, Thermo Fisher Scientific), and 75 µL of supernatant was transferred to a fresh glass vial for liquid chromatography-mass spectrometry (LC-MS) analysis. The quality control sample was prepared by mixing an equal aliquot of the supernatants from all of the samples. The ultra-performance liquid chromatography separation was carried out using a 1290 Infinity series System (Agilent Technologies (China) Co., Ltd., Beijing, China), equipped with a Kinetex C18 column (2.1 mm × 100 mm, 1.7 μm, Phenomen (Tianjin) Technology Dev, Ltd., Tianjin, China). The mobile phase A consisted of 40% water, 60% acetonitrile, and 10 mmol/L ammonium formate (Merck KGaA, Darmstadt, Germany). The mobile phase B consisted of 10% acetonitrile and 90% isopropanol, which was added with 50 mL 10 mmol/L ammonium formate for every 1,000 mL mixed solvent (Merck KGaA, Darmstadt, Germany). The analysis was carried out with elution gradient as follows: 0 to 12.0 min, 40%–100% B; 12.0 to 13.5 min, 100% B; 13.5 to 13.7 min, 100%–40% B; 13.7 to 18.0 min, 40% B. The column temperature was 45 °C. The auto-sampler temperature was 4 °C, and the injection volume was 0.5 µL (pos) or 4 µL (neg), respectively. The Triple TOF mass spectrometer (AB Sciex, MA, USA) was used for its ability to acquire MS/MS spectra on an information-dependent basis. In this mode, the acquisition software (Analyst TF 1.7, AB Sciex) continuously evaluates the full scan survey MS data as it collects and triggers the acquisition of MS/MS spectra depending on preselected criteria. In each cycle, the most intensive 12 precursor ions with intensity above 100 were chosen for MS/MS at a collision energy of 45 eV (12 MS/MS events with an accumulation time of 50 msec each). ESI source conditions were set as follows: Gas 1 as 60 psi, Gas 2 as 60 psi, Curtain Gas as 30 psi, Source Temperature as 600 °C, Declustering potential as 100 V, Ion Spray Voltage Floating as 5,000 V or –4,500 V in positive or negative modes, respectively.

On d 7 and 21, the same 6 dairy cows per group were randomly selected to collect rumen fluid. Original rumen content was collected before morning feeding via oral intubation (Wuhan Anscitech Farming Technology Co., Ltd., Wuhan, China). Approximately initial 50 mL was discarded to avoid saliva contamination and subsequent 50 mL from each cow was stored at −80 °C for 16S rRNA sequencing. For laboratory analysis, the pH was determined by a Sartorius PB-10 pH meter (Beijing Sartorius Stedim Biotech Co., Ltd., Beijing, China). The volatile fatty acid analysis was conducted according to the description of Kong et al. [23] combined with Agilent 6890 N gas chromatography (Agilent Technologies (China) Co., Ltd., Beijing, China). The total volatile fatty acid (TVFA) is the sum of individual volatile fatty acids. Volatile fatty acid was shown as the molar proportion and then we calculated the ratio of acetic acid to propionic acid (A:P).

The DNA of rumen fluid was extracted using the TIANMicrobe Magnetic Pathogen RNA Kit (TianGen Biotech (Beijing) Co., Ltd., Beijing, China) according to instructions. Then, the HiScript III 1st Strand cDNA Synthesis Kit (+ gDNA wiper, Vazyme Biotech Co., Ltd., Nanjing, China) was used to obtain cDNA by reverse transcription. DNA concentration and purity were monitored on 1% agarose gels and the qualified samples were used in the following analysis. The V3–V4 hypervariable region of the bacterial 16S rRNA gene was amplified with the primers 338F (5´-ACTCCTACGGGAGGCAGCAG-3´) and 806R (5´-GGACTACNNGGGTATCTAAT-3´) [25]. For each sample, an 8-digit barcode sequence was added to the 5´ end of the forward and reverse primers (provided by Allwegene Company, Beijing, China). The PCR was carried out on a Mastercycler Gradient (Eppendorf SE, Hamburg, Germany) using 25 µL reaction volumes, which contained 12.5 µL 2× Taq PCR MasterMix, 3 µL BSA (2 ng/µL), 1 µL forward Primer (5 µmol/L), 1 µL Reverse Primer (5 µmol/L), 2 µL template DNA, and 5.5 µL ddH_2_O. Cycling parameters were 95 °C for 5 min, followed by 28 cycles of 95 °C for 45 s, 55 °C for 50 s, and 72 °C for 45 s with a final extension at 72 °C for 10 min. The PCR products were purified using an Agencourt AMPure XP Kit (Beckman Coulter Inc., CA, USA). Deep sequencing was performed on the Miseq PE300 platform at Allwegene Company (Beijing, China).

On d 21, three dairy cows per group were selected randomly. The liver samples were obtained as described in a previous study [26]. Then, total RNA from liver tissue was prepared using TRIzol (Thermo Fisher Scientific), and we examined the quality and integrity as the previous description [26]. cDNA library preparation and sequencing were conducted according to the description by Ren et al. [27]. Then, the PCR products were purified and library quality was assessed on the Agilent Bioanalyzer 2100 system (Agilent Technologies (China) Co., Ltd., Beijing, China) and qualified by the ABI StepOnePlus Real-time PCR system (Thermo Fisher Scientific). Finally, the amplified library was sequenced on an Illumina HiSeq™ 2000 sequencing machine (Illumina, Inc., CA, USA). Six genes were selected randomly to verify the gene expression. The prime information was presented in Additional file 3. Lightcycer480 RT-PCR system (Roche company, Basel, Switzerland) and STBR®Premix Ex TaqTM II (Takara Biomedical Technology (Beijing) Ltd., Beijing, China) were used. The qPCR cycle included 94 °C for 2 min, 94 °C for 15 s, 56 °C for 15 s, 72 °C for 20 s, and 80 °C for 1 s and contained 45 cycles.

### Statistical analysis

For production performance, plasma biochemical indices, and rumen fermentation parameters, data were analyzed as a completely randomized design with repeated measures using the MIXED produce (SAS Institute Inc., Cary, NC, USA). The residual analysis was carried out to check the model assumptions. A normality check was conducted using the SAS Univariate procedure with Normal and Plot options. Group, time, and their interaction were considered as fixed effects, and cow within the group was considered as a random effect. The production performance indices were shown as average means of 3 d. When differences drawn from interactions or dietary treatments were detected, means separation was conducted using a Tukey adjustment for the probability. Significance was declared as *P* < 0.05 and trends were considered at 0.05 ≤ *P* < 0.10.

The data analysis for lipidomics was conducted as follows. The raw data files (.wiff format) were converted to files in mzXML format using the ‘msconvert’ program from ProteoWizard. Peak detection was first applied to the MS1 data. The CentWave algorithm in XCMS was used for peak detection. With the MS/MS spectrum, lipid identification was achieved through a spectral match using an in-house MS/MS spectral library (Igenecode Gene Technology Ltd., Beijing, China). A total of 13,457 and 14,491 peaks were extracted from positive and negative models. Missing value recording was conducted by half of the minimum value and normalization was conducted by internal standard method. Only peak intensive with more than 50% null value in a single group or all samples were removed. Principal component analysis was used via SIMCA software (V16.0.2 Sartorius Stedim Data Analytics AB, Umea, Sweden). Significantly different lipids (SDL) were filtered by *P* < 0.05 (student’s *t*-test) and variable importance in the production (VIP) > 1. Mantel analysis was used to calculate the relationship between plasma biochemical indices and SDL. Correlations were defined when *P*-values were *P* < 0.05.

The data analysis for the microbiome was conducted as follows. The raw data were first screened and sequences were removed from consideration if they (1) were shorter than 120 bp, (2) had a low-quality score (≤ 20), (3) contained ambiguous bases or did not exactly match primer sequences and barcode tags, and separated using the sample-specific barcode sequences. Qualified tags were denoised into amplicon sequence variants (ASV) using the unoise3 algorithm of Usearch (V10.0.240) software [28]. The BLAST tool was used to classify all sequences into different taxonomic groups against the Silva138 database [29]. QIIME (V1.8.0) was used to calculate the richness and diversity indices based on the ASV information. Principal coordinates analysis (PCoA) was analyzed by R (v3.6.0) based on the ASV information from each sample [30]. To examine the similarity and structure of communities between different samples, a Simper analysis was conducted and heatmaps were generated by Majorbio Cloud [31]. Wilcoxon rank-sum test was used to analyze differences in diversity indices and genera. The related column diagrams were generated by GraphPad Prism 7. The correlation analyses between genera and plasma biochemical indices, genera and genera were calculated and plotted by Majorbio Cloud [31].

The data analysis for transcriptomics was conducted as follows. The image dataset was converted to raw data by Base Calling. RNA-Seq reads were first performed for quality control to remove low-quality sequences including (1) removing reads with more than 5% ‘N’ bases, (2) 20% QA ≤ 10 bases, and (3) joint pollution. HISAT was used to map clean reads to reference genome [32] and RSEM was used to calculate the gene expression by the number of fragments per kilobase of exon model per million mapped fragments (FPKM) [33]. Differential expression analysis of two groups was performed using the DEseq2 and the criteria were fold change (FC) ≥ 2 or ≤ 0.5 and adjusted *P* ≤ 0.05. Kyoto Encyclopedia of Genes and Genomes (KEGG, http://www.genome.jp/kegg/), Gene Ontology (GO, http://www.geneontology.org/), and String (https://string-db.org/) database were used to enrich pathways and protein-protein interaction network.

## Results

### Microbiota analyses

**Fig. 1:**
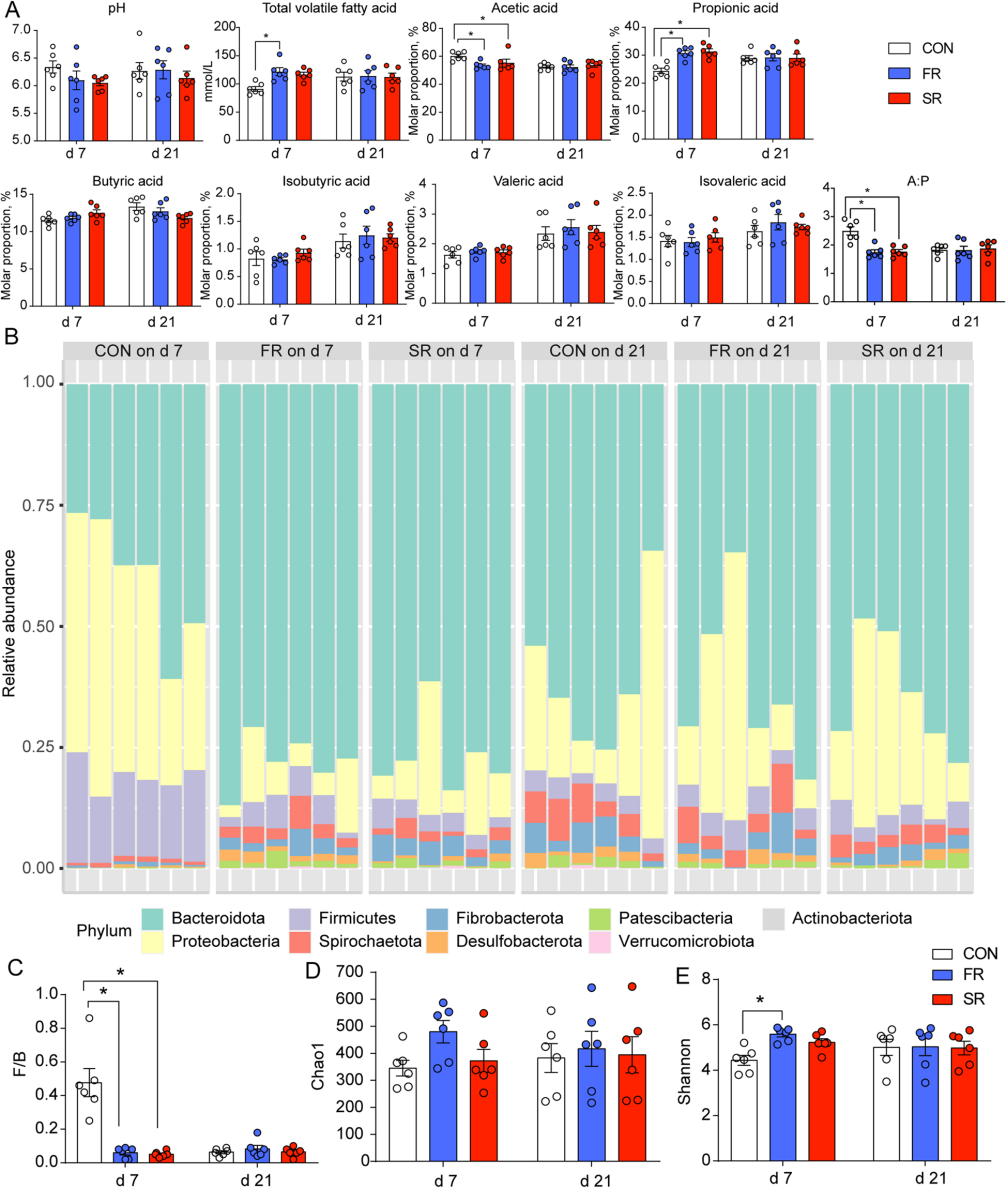
Effects of rumen microbiota transplantation (FR), sterile rumen fluid transplantation (SR) on rumen fermentation and microbial diversity. The dairy cows in the CON group received 10 L saline daily. **A** The rumen fermentation parameters and individual volatile fatty acids are shown as molar proportions. **B** The composition of relative abundance of microbiota at the phylum level. **C** The ratio of Firmicutes to Bacteroides. **D** Chao1 index calculated at the ASV level. **E** Shannon index calculated at the ASV level. Mann–Whitney U test was used for variation analysis. ^*^0.01 ≤ *P* < 0.05; ^**^0.001 ≤ *P* < 0.01; ^***^0.0001 ≤ *P* < 0.001. Data are shown as mean ± SEM. *n* = 6

The RMT was conducted during the first 3 d after postpartum. Hence, we analyzed the microbial diversity and rumen fermentation parameters. The differences between d 7 and 21 may show the colonization effect and sustaining effect. The pH value was not affected and cows in the FR group had a lower TVFA concentration, molar proportion of acetic acid, A:P, and higher molar proportion of propionic acid (Fig. 1A). The cows in the SR group also had a lower molar proportion of acetic acid, A:P, and a higher molar proportion of propionic acid (Fig. 1A). Then, 16S rRNA sequencing shows that the most abundant phyla (average relative abundance > 1%) on d 7 in the CON group are Proteobacteria (40.97%), Bacteroidetes (39.92%) and Firmicutes (17.33%), while the most abundance phyla transform into Bacteroides (68.48%), Proteobacteria (17.74%), Firmicutes (4.18%), Spirochaetes (3.75%), Fibrobacteres (3.28%), and Patescibacteria (1.16%) on d 7 in the FR and SR groups and on d 21 in the CON, FR, and SR groups (Fig. 1B). The ratio of Firmicutes to Bacteroides (F/B) is a classical index to evaluate the composition or type of microbiota (Fig. 1C). On d 7, F/B in the CON group is significantly higher than FR and SR groups (*P* < 0.05). Then, there is no difference on d 21 (*P* > 0.05). For alpha diversity, the Shannon index of the CON group on d 7 was lower than the FR group (*P* < 0.05) and had no difference on d 21 (*P* > 0.05) in Fig. 1E.

**Fig. 2:**
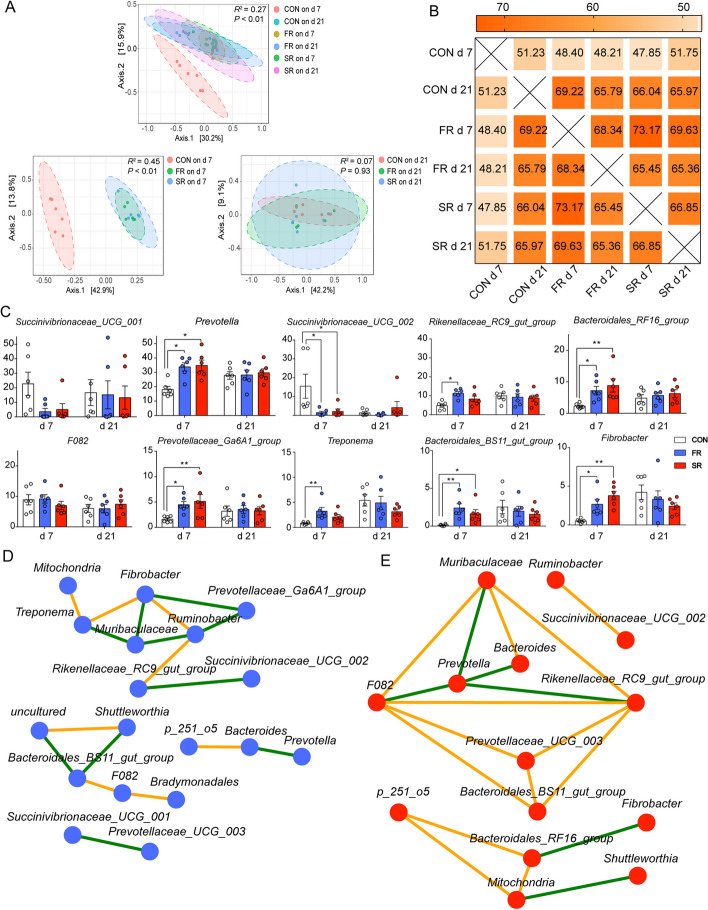
The similarity among different groups. **A** Principal coordinates analysis (PCoA) based on Bray-Curtis distance matrix and ASV level. PERMANOVA analysis was used to group similarities. **B** Similarity percentage (SIMPER) analysis among different group. **C** The top 10 genera that accounted for a variance was presented. Mann–Whitney U test was used for variation analysis. **D** and **E** Network of co-occurring genera in the SR (**D**) and FR group (**E**). The blue nodes indicate genera in the FR group and the red nodes indicate genera in the SR group. The color edges stand for strong (Spearman’s correlation coefficient < −0.5 or > 0.5) and significant (*P* < 0.05) correlations between genera, including positive correlations (orange) and negative correlations (green). ^*^0.01 ≤ *P* < 0.05; ^**^0.001 ≤ *P* < 0.01; ^***^0.0001 ≤ *P* < 0.001. Data are shown as mean ± SEM. *n* = 6

The beta diversity analysis is visualized using PCoA based on the Bray-Curtis distance matrix and ASV level, and further analysis by PERMANOVA (Fig. 2A) shows that the microbiota on d 7 in the CON group is separating from FR and SR groups (*P* < 0.05), and this difference disappears on d 21 (*P* > 0.05). There is no separation between the FR group and the SR group on any day (Fig. 2A). Furthermore, we used Simper analysis to compare the similarity of microbiota and calculate the variance contribution. The results in Fig. 2B show that the similarities between the CON group and FR or SR group on d 7 were 48.40% and 47.85%, and the similarity between d 7 and 21 in the CON group was 51.23%. Interestingly, the similarities were increased in the FR (68.34%) and SR groups (66.85%) between d 7 and 21. The microbiota on d 7 in the FR group showed the highest similarity when compared with the SR group (73.17%). Then, we show the top 10 genera that contributed to the variance mostly among different groups, and the variation contribution was presented in Additional file 4. The genera including *Prevotella*, *Rikenellaceae_RC9_gut_group*, *Prevotellaceae_Ga6A1_group*, and *Bacteroidales_BS11_gut_group*, *Bacteroidales_RF16_group* belonging to Bacteroides, and *Fibrobacter* belonging to Fibrobacteres were higher on d 7 in the CON group when compared with FR and SR groups in Fig. 2C (*P* < 0.05). On d 21, these genera were not significantly different (*P* > 0.05). *Succinivibrionaceae_UCG-001* and *Succinivibrionaceae_UCG-002* belong to Proteobacteria on d 7 in the CON group were significantly lower than FR and SR groups (*P* < 0.05) and the two genera were not changed in d 21 (*P* > 0.05).

Although the PCoA plot and similarity analysis (Fig. 2A and B) indicated the similarity between the SR group and the FR group on d 7 was highest in this study, further analysis of the microbial correlation network at the genus level is present in the Fig. 2D and E to investigate the different interaction. The genera with relative abundance > 1% were used in this analysis. The network of the FR group consisted of 18 nodes and 18 edges, including 8 positive correlations and 10 negative correlations (Fig. 2D). The network of the SR group consisted of 14 nodes and 18 edges, including 12 positive correlations and 6 negative correlations. The only same co-occurring genera in the FR and SR groups is the positive correlation between *F082* and *Bacteroidales_BS11_gut_group* (Fig. 2E).

### Production performance and plasma biochemical parameters

**Fig. 3:**
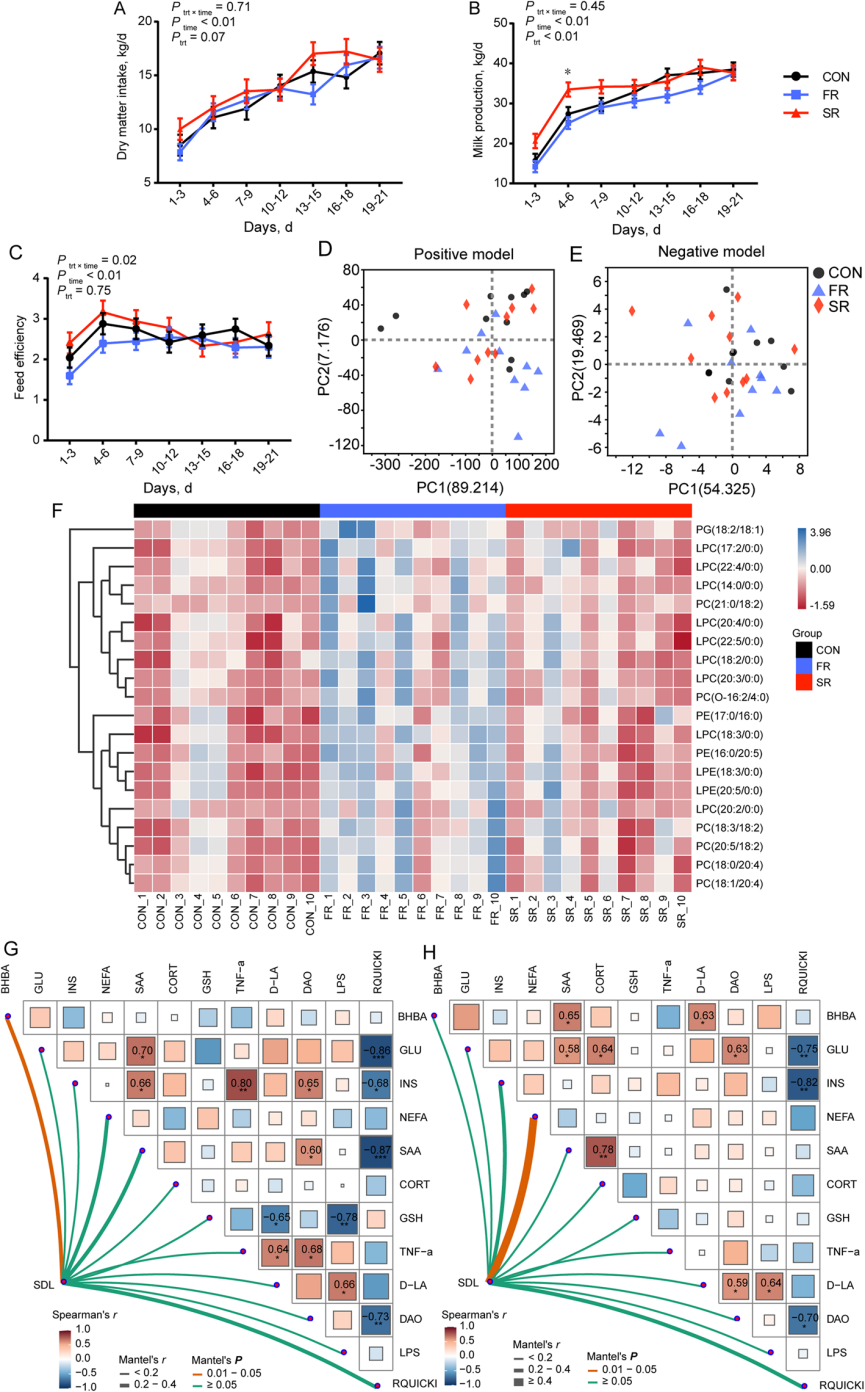
Effects of rumen microbiota transplantation (FR), sterile rumen fluid transplantation (SR) on production performance, plasma lipidome in postpartum dairy cows. The dairy cows in the CON group received 10 L saline daily. **A–C** Average dry matter intake (**A**), milk production (**B**), and feed efficiency (**C**) at 3-d intervals during postpartum 21 d including 7-time points. **D** and **E** The lipidome profiles of plasma visualized by score plots of principal component analysis (PCA) in the positive model (**D**) and negative model (**E**). **F** Heatmap plot used to show the relative intensive of SDL with FC > 2 or < 0.5. The colors of heatmap cells indicate the level of lipids across different samples. **G** and **H** Mantel analysis between significantly different lipids matrix and plasma parameter matrix. Color gradients indicate Spearman’s correlation coefficients. Edge width corresponds to the Mantel’s *r* value and edge color denotes the statistical significance. ^*^0.01 ≤ *P* < 0.05; ^**^0.001 ≤ *P* < 0.01; ^***^0.0001 ≤ *P* < 0.001. Data are shown as mean ± SEM. *n* = 10

The DMI, milk production, and feed efficiency were the most common indices to evaluate physiological status (Fig. 3A–C). The production performance data were significantly affected by the time effect (*P* < 0.05). The average milk production in the SR group was significantly higher than the CON group, and both CON and SR groups were significantly higher than the FR group (Additional file 5, *P* < 0.05). However, there are no significant differences in DMI and feed efficiency between the CON group and FR or SR group (Fig. 3A and C; Additional file 5).

We also detected the biochemical indices in plasma to reveal the changes in glucolipid metabolism (Table 1). Because the disorder of glucolipid metabolism during the postpartum period leads to diverse diseases. First, the BHBA concentrations in the FR group on both d 7 and 21 were lower than those in the CON group (*P* < 0.05). No significant differences were found in the other glucolipid metabolism and immune indices (*P* > 0.05). The GSH-Px concentration in the SR group was higher than the CON group on d 7 and the same with the CON group on d 21 (*P* < 0.05). The TNF-α concentration in the SR group was higher than the CON group on d 21 (*P* < 0.05). The D-LA and DAO concentrations in the SR and FR groups were significantly higher than the CON group on d 21 and the other bacterial component, LPS in the two groups was lower than the CON group on d 7 and 21 (*P* < 0.05).

**Table 1:** Effects of rumen microbiota transplantation (FR), sterile rumen fluid transplantation (SR) on plasma biochemical indices in postpartum dairy cows^1^

Items	7 d			21 d			SEM	*P-*value		
	CON	FR	SR	CON	FR	SR		Treatment	Time	Interaction
Glycolipid metabolism										
BHBA, mmol/L	1.47^A^	1.34^C^	1.45^A^	1.59^A^	1.49^B^	1.51^AB^	0.033	0.01	< 0.01	0.27
NEFA, μmol/L	93.71	95.37	98.04	99.73	94.54	99.88	3.494	0.54	0.35	0.52
GLU, mmol/L	3.18	3.19	2.68	3.30	3.34	2.74	0.207	0.05	0.30	0.92
TG, mmol/L	0.04	0.03	0.04	0.04	0.05	0.06	0.012	0.44	0.20	0.80
TC, mmol/L	2.02	2.43	2.00	3.28	3.60	3.39	0.295	0.48	< 0.01	0.80
INS, mIU/L	13.96	15.72	12.34	16.17	14.14	13.22	1.233	0.14	0.59	0.23
Cort, ng/mL	17.86^AB^	17.12^B^	17,51^AB^	19.65^A^	17.68^AB^	20.08^A^	0.641	0.03	< 0.01	0.25
RQUICKI^2^	0.54	0.52	0.58	0.51	0.53	0.56	0.017	0.07	0.20	0.28
Immune indices										
IgA, μg/mL	93.84	96.48	97.04	104.14	104.45	100.99	3.334	0.89	0.01	0.61
IgG, mg/mL	11.20	10.56	10.64	11.02	12.25	11.26	0.476	0.60	0.07	0.14
IgM, mg/mL	0.65	0.63	0.62	0.66	0.68	0.68	0.018	0.95	< 0.01	0.09
Antioxidant indices										
GSH-Px, μmol/L	6.50^B^	6.32^B^	8.27^A^	6.42^B^	5.10^C^	6.81^B^	0.570	0.01	0.05	0.43
SOD, U/mL	73.23	68.66	71.05	70.02	70.71	73.47	2.208	0.53	0.79	0.31
MDA, nmol/mL	1.23	1.24	1.31	1.30	1.28	1.32	0.046	0.34	0.21	0.74
T-AOC, U/mL	10.36	10.64	10.13	10.41	11.51	10.41	0.638	0.37	0.43	0.79
Microbial compositions										
D-LA, μmol/L	6.19^A^	5.87^AB^	5.60^B^	5.70^B^	6.42^A^	6.46^A^	0.164	0.49	0.03	< 0.01
DAO, U/mL	3.37^C^	3.53^BC^	3.37^C^	3.23^C^	4.30^A^	3.87^B^	0.132	< 0.01	< 0.01	0.01
LPS, EU/mL	0.83^A^	0.72^B^	0.73^B^	0.80^A^	0.72^B^	0.76^BC^	0.036	0.02	0.95	0.39
Other indices										
SAA, µg/mL	26.33^BC^	25.41^C^	24.31^C^	29.56^AB^	26.16^BC^	30.85^A^	1.027	0.05	< 0.01	0.02
TNF-α, ng/L	131.08^B^	150.80^A^	135.77^B^	139.46^B^	135.94^B^	156.05^A^	4.356	0.05	0.20	< 0.01

*BHBA* β-hydroxybutyric acid, *NEFA* Nonesterified fatty acid, *GLU* Glucose, *TG* Triglyceride, *TC* Total cholesterol, *INS* Insulin cortisol, *IgA* Immunoglobulin A, *IgG* Immunoglobulin G, *IgM* Immunoglobulin M, *GSH-Px* Glutathione peroxidase, *SOD* Superoxide dismutase, *MDA* Malondialdehyde, *T-AOC* Total antioxidant capacity, *D-LA* D-lactic acid, *DAO* Diamine oxidase, *LPS* Lipopolysaccharide, *SAA* Serum amyloid A, *TNF-α* Tumor necrosis factor-α

^1^The dairy cows in the CON group received 10 L saline daily

^2^Revised quantitative insulin sensitivity check index (RQUICKI) = 1/[log(GLU) + log(INS) + log(NEFA)] [24]

^A–C^Different superscript letters in each indicator represent a significant difference (*P* < 0.05)

### Plasma lipidome

Since body fat mobilization and milk fat synthesis occur after parturition, and the role of lipids as bioactive signaling molecules capable of insulin resistance, immunoregulation, and inflammatory response, we investigated whether lipidome was changed by RMT. Visually, the principal component analysis (PCA) plots in Fig. 3D and E show no clear distinction among different groups. Furthermore, variation analysis was conducted based on VIP value and *P* value (VIP > 1; *P* < 0.05). A total of 191 and 27 lipids were significantly changed in the FR and SR groups when compared with the CON group (Additional files 6 and 7). Then, FC was further used to filtrate vastly different lipids (FC > 2 or < 0.5). No lipids showed large different changes between CON and SR groups and there were no significantly decreased lipids in the FR group when compared with the CON group. Figure 3F shows a total of 20 lipids extremely increased in the FR group when compared with the CON group and increased lipids include 9 lyso-phosphatidylcholines (LPC), 2 lyso-phosphatidylethanolamines (LPE), 6 phosphatidyl cholines (PC), 2 phosphatidylethanolamines (PE), and 1 phosphatidylglycerol (PG). Furthermore, a total of 5 lipids in Fig. 3F contain C18:2, and 3 lipids contain C18:3, C20:4, and C20:5.

The Mantel test was used to investigate the correlation between plasma parameters and SDL (Fig. 3G and H). Firstly, the correlation analysis found that RQUICKI was negatively correlated with GLU, INS, and DAO in Fig. 3G and H (*P* < 0.05). The LPS, the membrane component of bacteria, was positively correlative with D-LA and GLU was positively correlative with SAA (*P* < 0.05). Finally, the mental test found that the SDL matrix is significantly correlated with BHBA concentration in plasma within the CON and FR groups (Fig. 3G, *P* < 0.05) and SDL matrix is significantly correlated with NEFA concentration within the CON and SR groups (Fig. 3H, *P* < 0.05).

### Correlation analysis between genera and plasma biochemical indices

**Fig. 4:**
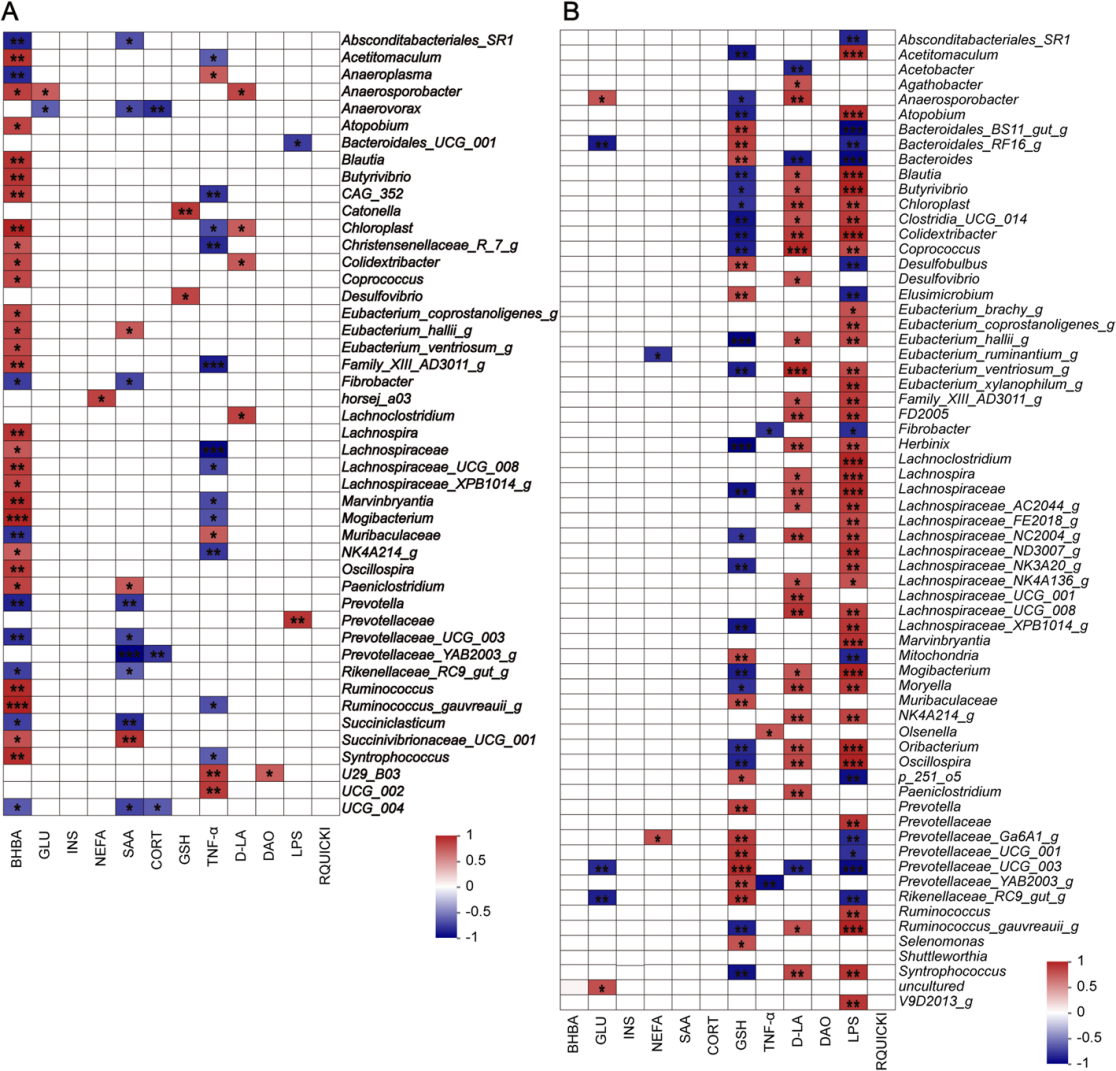
Spearman correlations between genera and significantly different plasma parameters including CON and FR groups (**A**) and CON and SR groups (**B**). Only significant correlations are presented. ^*^0.01 ≤ *P* < 0.05; ^**^0.001 ≤ *P* < 0.01; ^***^0.0001 ≤ *P* < 0.001. *n* = 6

The above analyses found the fresh and sterile RFM significantly accelerated the maturity of rumen microbiota in different manners (Fig. 2D and E). Hence, we used correlation analysis between genera and significantly changed plasma parameters (Fig. 4). The correlation plots show that the relative abundances of *Prevotella*, *Rikenellaceae_RC9_gut_group*, *Fibrobacter*, and *Prevotella_UCG_003* were negatively correlated with BHBA concentration (Fig. 4A). The majority of genera that were positively correlated with BHBA, belong to Firmicutes, such as *Acetitomaculum*, *Anaerosporobacter*, and other 22 genera (Fig. 4A). When the data set included CON and SR groups, the results showed that GSH, D-LA, and LPS were correlated with most of the genera (Fig. 4B). GSH concentration was positively correlated with genera belonging to Bacteroides, such as *Prevotella*, *Prevotellaceae_Ga6A1_group*, *Prevotellaceae_UCG-001*, and *Prevotellaceae_UCG-003* (Fig. 4B). Conversely, D-LA and LPS concentrations were positively correlated with genera belonging to Firmicutes (Fig. 4B).

### Liver transcriptome

**Fig. 5:**
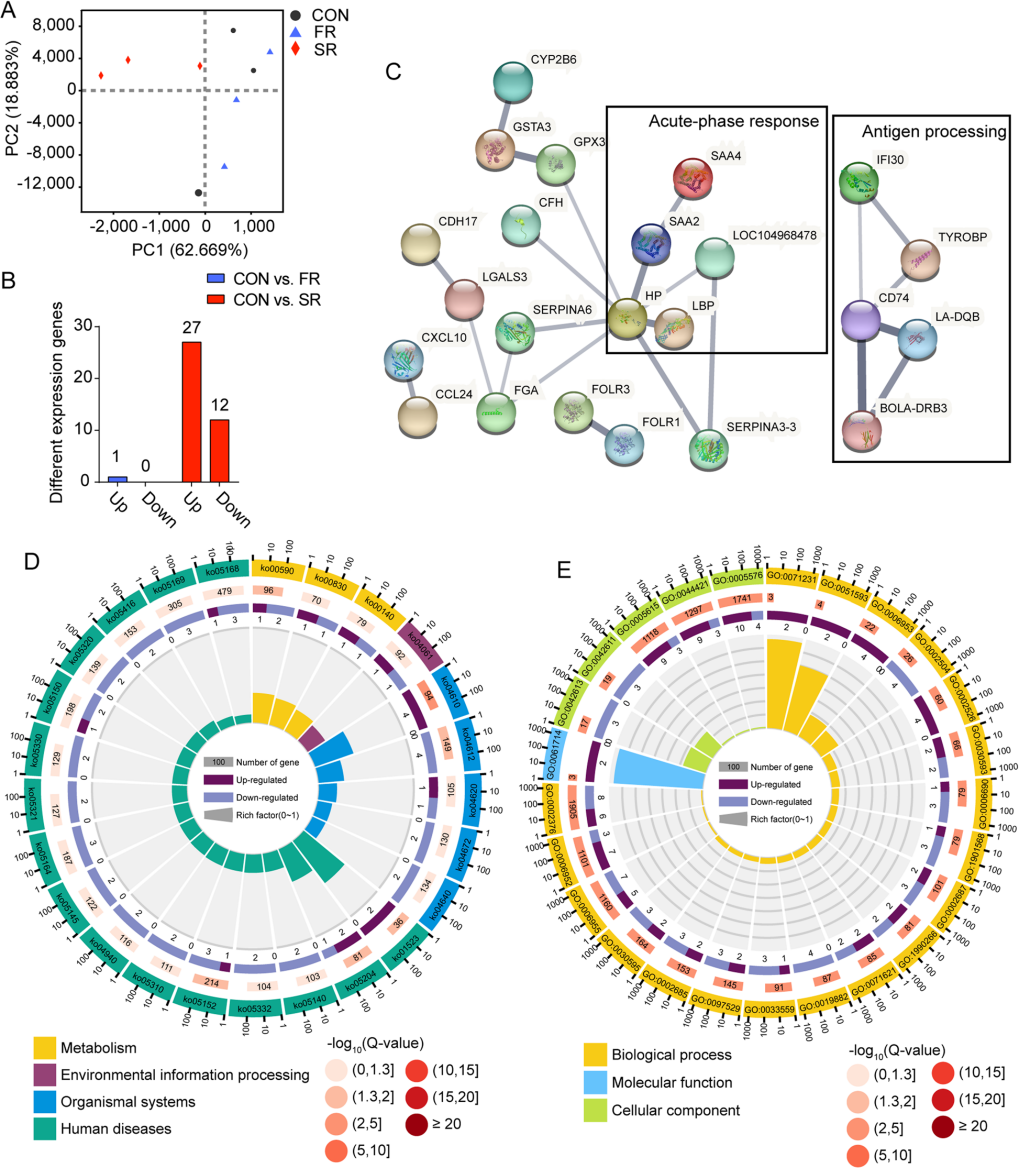
Effects of rumen microbiota transplantation (FR), sterile rumen fluid transplantation (SR) on gene expression in the liver. The dairy cows in the CON group received 10 L saline daily. **A** Principal component analysis (PCA) of gene expression in the liver on d 7. **B** Number of different expression genes on d 7 in the FR and SR groups when compared with the CON group. **C** Protein-protein interactions of different expression genes between CON group and SR group using STRING database. The line thickness indicates the strength of data support. The black box indicates the genes in the box contribute to the same function. **D** KEGG enrichment of different expression genes on d 7 between the CON group and SR group. The first lap shows the top 25 KEGG terms and the number of genes corresponds to the outer lap. The second lap indicates the background genome and *P* value for enrichment of the differential expression genes. The third lap indicated the ratio and number of the upregulated genes (deep purple) and downregulated genes (light purple). The fourth lap shows the enrichment factor of each KEGG term. **E** GO enrichment of differential expression genes on d 7 between the CON group and SR group. The legend was similar to **D** as mentioned above. *n* = 3

The gene expression profile of the liver regulates the glucolipid metabolism of dairy cows during the transition period and the plasma parameters mentioned above indicate the gene expression profile may be changed by treatment. Hence, transcriptomics was used to describe the changes on d 7. First, the PCA plot in Fig. 5A shows the samples in the SR group were clustered and far away from the CON group and FR group. Then, we identified 1 gene, *ZNF606*, that was significantly changed in the FR group when compared with the CON group, and another 39 genes were changed in the SR group when compared with the CON group (adjusted *P* < 0.05). In Fig. 5B, 12 genes were significantly downregulated and another 27 genes were significantly upregulated in the liver of the SR group. Then, we input the different expression genes (DEG) into the String database and the edges between genes indicated the protein expressed by DEG jointly contribute to a shared function (Fig. 5C). The network composed of *SAA4, SAA2, HP, LBP*, and *LOC104968478* was associated with acute-phase response and the network composed of *IFI30, TYROBP, CD74, LA-DQB*, and *BOLA-DRB3* was associated with antigen processing. Finally, KEGG enrichment and GO enrichment were conducted (Fig. 5D and E). Metabolism pathways including arachidonic acid metabolism (ko00590), retinol metabolism (ko00830), and steroid hormone biosynthesis (ko00140) were significantly changed (*P* < 0.05). The complement and coagulation cascade pathway (ko04610) was upregulated and antigen processing and presentation (ko04612) was downregulated in the SR group when compared with the CON group (*P* < 0.05). GO enrichment found the acute-phase response (GO:0006953) and acute inflammatory response (GO:0002526). Several pathways regarding immunoreaction, such as immune response (GO:0006955), positive regulation of leukocyte migration (GO:0002687), and immune system process (GO:0002376), were also significantly changed. Finally, the validation of genes was presented in Additional file 8. The gene expression levels expressed by RNA-Seq and RT-PCR showed the same trends among the three groups indicating the RNA-Seq results were reliable.

## Discussion

The microbiota in the rumen of dairy cows includes diverse and high-density microbes. Due to dietary transition and calving stress, the microbiota of the rumen in the postpartum period also reshapes the composition within about 21 d. Few studies have investigated the effects and applications of RMT. Hence, we conducted RMT from peak-lactating dairy cows to fresh cows in the form of fresh rumen fluid and inactivated rumen fluid to expound the role of rumen microbiota in the postpartum period. The most common methods of rumen fluid collection include donors equipped with a stomach tube, abattoir slaughter, or at post-mortem [16]. In our study, a total of 12 peak-lactating dairy cows equipped with rumen fistula were used to supply rumen fluid to obtain homogeneous rumen fluid.

The first aim of this study is to determine whether RMT can alter the rumen environment. The 16S rRNA sequencing showed that the bacterial composition in the CON group varied from d 7 to 21. Many longitudinal studies have investigated the dynamic changes of rumen microbiota during the transition period (from 3 weeks before to 3 weeks after parturition). The F/B has traditionally been considered a biomarker for the metabolic potential of the microbiota and is known to modulate host metabolism [34]. A repeated measurement from Derakhshani et al. [10] found the relative abundance of Bacteroidetes was increased and the relative abundance of Firmicutes was decreased after parturition, which was in agreement with our results. However, the dominating phyle differs in their relative abundance from study to study over the transition period [10, 12, 35]. This may be explained by differences in diet or environmental factors [36, 37]. Anyway, the phylum compositions and F/B after RMT or sterile RMT treatments on d 7 were similar to the composition in the CON group on d 21 in our study, which meant that the treatments successfully shortened the original transition period from 21 d to 7 d or even shorter.

We found the genera that accounted for variance among the three groups mostly showed a similar trend in the SR and FR groups when compared with the CON group. It has been reported that *Prevotella* spp., which is primarily known for its amylolytic and proteolytic properties, has been extensively reported during adaptations of the ruminal microbiota to high-concentrate diets [13]. In our study, *Prevotella* spp. accounted for the microbiota mostly, and genera including *Prevotella* and *Prevotella_Ga6A1_group* were increased by fresh RMT and sterile RMT treatment on d 7, which was consistent with the increase of molar proportion of propionic acid.

Succinivibrionaceae is also considered a core member of the ruminal microbiome associated with high-concentrate diets [38] and members of the Succinivibrionaceae family were negatively associated with methane emission because its members mainly produce succinate, thereby trapping metabolic hydrogen rather than releasing hydrogen [39, 40]. *Succinivibrionaceae_UCG-001* was enriched in the dairy cow feeding a high-energy (high-concentrate) diet [41]. Zhu et al. [13] found that genera including *Ruminobacter Succinivibrio* and *Unclassified Succinivibrionaceae*, which from the Succinivibrionaceae family, were increased together after parturition. Furthermore, *Bacteroidales_BS11_gut_group*, *Bacteroidales_RF16_group*, and *Fibrobacter* were responsible for fermenting hemicellulose or cellulose to short-chain fatty acids [42, 43]. Studies mentioned above were inconsistent with the high-concentrate diet used in our study and the transition trend of these genera, in which *Succinivibrionaceae_UCG-001* and *Succinivibrionaceae_UCG-002* were both decreased and *Bacteroidales_BS11_gut_group*, *Bacteroidales_RF16_group*, and *Fibrobacter* were increased after treatments. The change curve of the relative abundance in the transition period may explain these incompatible results. Zhu et al. [13] investigated the changes in the ruminal microbiome from 3 weeks before parturition to 4 weeks after parturition and found that the relative abundance of major bacterial taxa changed up and down. For example, several genera display the tendency to rise at the beginning (weeks 1–3) and decline late, including *Prevotella*, *Unclassified Succinivibrionaceae*, and so on. The relative abundance of *Fibrobacter* was increased and reached the maximum at week 2 and then decreased gradually [13]. Similar results also reappear in another longitudinal study [44], indicating the relative abundance of genera that turn into plateaus within different durations. Hence, it is necessary to investigate the causal relationship between host metabolism and the pattern of the ruminal microbiome.

Interestingly, introducing inactivated rumen fluid was also an effective way to mature rumen microbiota like fresh rumen fluid. The sterile microbial mixture contains microbial debris, metabolic products, exfoliated rumen cells, and so on [45]. A cross-incubation experiment combining metabolites and microbiota from different time points after feeding indicated that methane production in the evening was increased than morning in vivo and further rumen fluid metabolites in the evening promoted higher methane production than the metabolic environment in the morning in vitro [46]. The results highlight the niche modification as a selective deterministic process that shaped microbiota community assembly via environmental filtering. Moreover, the RMT conducted in young ruminants indicated that fresh rumen fluid was effective in accelerating rumen development and had the potential implementation of early weaning strategies, which the sterilized rumen fluid did not have any benefit [18, 19]. We speculated the original microbiota of the receptor may influence the effects of RMT. The previous study in our team found that different pretreatments for FMT significantly affected the colonization of microbes and antibiotic pretreatment enhances gut microbiota reprogramming by promoting xenomicrobiota colonization [47]. Hence, we speculated that the rare microbes in young ruminants may promote microbial colonization, while the sterilized rumen fluid may play as a filter to select the existing abundance of microbes. Furthermore, Ott et al. [48] transferred sterile fecal filtrate for treating patients with *Clostridium difficile* infection (CDI) and found that the microbiota composition was restored and the treatment eliminated symptoms of CDI for a minimum period of months. A key advantage of sterile RMT is the avoidance of all risks inherent to the transfer of living microbes [49, 50]. This could suggest a potential for sterile RMT to be applied to perinatal care because of the higher sensibility of transition dairy cows for pathogenic bacteria [51]. However, the further co-occurring network found the interactions between genera were different in reconstructing the ruminal microbiome by fresh RMT and sterile RMT. A further longitudinal investigation by detecting microbiota and metabolome at multiple time points may reveal the potential mechanism.

Our study is the first experiment to investigate RFM during the transition period. Because of the time limit and invasiveness, we did not empty and transplant the whole rumen content instead of introducing the new microbiota. Mu et al. [52] transplanted whole rumen content from healthy dairy cows to recipients with subacute ruminal acidosis and found the microbiota rebuild their rumen bacterial homeostasis quickly within 4–6 d after RMT. Another study only transplanted a quarter of the total rumen content to the recipient and found the rumen microbiota rebuild homeostasis within 7 d [53]. Although the transplant volume and method used in our study were different from these studies, the similarity between d 7 and 21 in the FR and SR groups indicated the rumen microbiota of recipients also rebuilt their homeostasis within 7 d.

After calving, the energy intake was not enough to meet the lactating requirement and body fat mobilization was induced by negative energy balance (NEB) [54]. There was no doubt that the DMI and milk production increased gradually after postpartum (Fig. 3A and B). Improving DMI is the most useful way to solve NEB, while the treatments in our study did not affect DMI and feed efficiency. The primary study showed that dairy cows following surgical repair of left-displaced abomasum receiving 10 L rumen fluid could increase DMI and milk yield, and decreased BHBA concentration [17]. Another investigation using cows suffering from indigestion found that RMT could improve feed intake [55]. These results indicated the effects of RMT on the DMI are dependent on the physiological states of the host. Dairy cows after the postpartum period suffer from the metabolic stress of lactation, which is different from disease states and it may contribute to the unchanged DMI.

Sterilized rumen fluid improved average milk production. On the one hand, we speculated that sterile rumen fluid could reduce the competition and energy consumption by the bacteria itself compared with FR and CON groups. It has been clarified that specific gut bacteria can consume dietary substrates resulting in a loss in the remaining nutrients which, in turn, can reduce the amount of nutrients that can be absorbed by the host [56]. On the other hand, rumen fluid acts as a nutritional supplement when the contents in microbes are released by sterile technology. This nutritional supplement may support the increase of milk production under the same degree of adipose mobilization (plasma NEFA concentration). The gastrointestinal tract microbial metabolites generally contain VFA, lactate, bacteriocins, and so on [57]. A previous study found that lactate concentration was significantly increased and VFA concentrations were not changed in sterilized rumen fluid when compared with fresh rumen fluid [19]. The unchanged rumen pH indicated the lactate may be taken by microbes.

Excessive lipolysis along with ketogenesis after the postpartum period is the reflection of high-yielding dairy cows [58]. We found fresh rumen fluid alleviated NEB and the decreased BHBA concentration in the FR group proved this opinion. We now know that in addition to energy metabolism, lipidome is important in regulating glucose homeostasis, insulin sensitivity, oxidative stress, inflammation, and immune function [4, 59, 60]. In our study, the lipidome was extensively affected by fresh RMT and the mantel analysis found the matrix including significantly different lipids was correlated with BHBA concentrations. Further filtration using FC (> 2 or < 0.5) found PC, LPC, and LPE were enriched and most lipids contained long-chain polyunsaturated fatty acids, such as C18:2, C20:4, and C20:5. In transition cows, PC concentrations are low in postpartum cows [61]. Hence, the essential requirement of PC synthesis for very low-density lipoprotein secretion may partially explain the lower milk production and plasma BHBA concentration because of transferring triglyceride from the liver to adipose tissue. In addition, the changes in the composition of plasma lipids could be driven by the alternations in fatty acids (FA) of adipose tissue as well as the direct incorporation from dietary FA. Transition dairy cows are characterized by increased concentrations of palmitic, stearic, oleic, and linoleic acids and decreased concentrations of long-chain polyunsaturated FA (PUFA) in serum [62]. A previous study also indicated that long-chain PUFA depletion was evident [62], which implied that hepatic membrane fluidity and condition decreased around parturition. The PUFA status of ruminants is notoriously precarious because ruminal biohydrogenation of dietary PUFA limits the supply for absorption [63]. Hence, increases in PC and PUFA indicated that fresh RMT may provide a stable way to alleviate fat mobilization and hepatic metabolism stress.

Differences between CON and SR groups were associated with acute-phase response, acute inflammatory response, immune response, and antigen processing in the liver when we enriched the differentially expressed genes into several databases. Horst et al. [64] reviewed the influence of immune activation and inflammation on transition dairy cows and concluded that almost all transition dairy cows, even healthy cows experience inflammation and immune activation which is accompanied by large changes in energetic and mineral metabolism to support the nutrient requirements of immune cell. We assumed that bacterial components including D-LA, DAO, and LPS in a sterile microbial mixture might take charge of these processes. Because LPS-treated cows in mid-lactation increased inflammatory cytokines, SAA, and haptoglobin in the liver [65]. However, LPS concentration and plasma biomarkers of the gut barrier (D-LA, DAO) were decreased or not affected at d 7 in the SR and FR groups. Given the complicated contents of sterile RMT and increases in D-LA and DAO concentrations at d 21, the adverse impacts on liver function should be explained by further investigation.

## Conclusions

This study demonstrated that both fresh RMT and sterile RMT successfully promoted the transition process of the ruminal microbiota from peripartum to postpartum within 7 d and promoted the production of propionic acid. This intervention of rumen bacterial composition had no benefit on DMI recovery and feed efficiency, supporting that inoculating rumen fluid may not be one of the routine procedures of postnatal care for Holstein dairy cows. However, fresh rumen fluid may have the potential to treat ketosis by improving the transport of NEFA from the liver to adipose tissue and causing positive effects on plasma BHBA concentration. Further research is needed to investigate the potential role of RMT on glycolipid metabolism in ketosis cows. Moreover, the metabolite composition of rumen fluid is also necessary to be further investigation.

### Supplementary Information

**Additional file 1: Table S1.** Diet ingredients and chemical composition of TMR diet.

**Additional file 2: Fig. S1.** Rumen microbiota transplantation process.

**Additional file 3: Table S2.** Primer sequences of validation genes.

**Additional file 4: Table S3.** Top 10 genera contributing to variance mostly among different groups.

**Additional file 5: Table S4.** Effects of fresh and sterile rumen microbiota transplantation on milk production and dry matter intake.

**Additional file 6: Table S5.** Significantly different plasma lipids between the CON group and FR group.

**Additional file 7: Table S6.** Significantly different plasma lipids between the CON group and SR group.

**Additional file 8: Fig. S2.** Validation of RNA-Seq by RT-PCR.

## Data Availability

The raw data from 16S rRNA and RNA-Seq were both deposited in the SRA database of NCBI and the project number was PRJNA976096 (Rumen microbiota transplantation and transition dairy cow microbiota regulation).

## Abbreviations

ASV: Amplicon sequence variants
A:P: The ration of acetic acid to propionic acid
BHBA: β-hydroxybutyric acid
CDI: *Clostridium* *difficile* infection
Cort: Cortisol
DAO: Diamine oxidase
DEG: Different expression genes
D-LA: D-lactic acid
DMI: Dry matter intake
F/B: Ratio of Firmicutes to Bacteroides
FC: Fold change
FMT: Fecal microbiota transplantation
FPKM: Million mapped fragments
GLU: Glucose
GO: Gene Ontology
GSH-Px: Glutathion peroxidase
IgA: Immunoglobulin A
IgG: Immunoglobulin G
IgM: Immunoglobulin M
LC-MS: Liquid chromatography-mass spectrometry
LPC: Lyso-phosphatidylcholines
LPE: Lyso-phosphatidylethanolamines
LPS: Lipopolysaccharide
MDA: Malondialdehyde
NEB: Negative energy balance
NEFA: Nonesterified fatty acid
PC: Phosphatidyl cholines
PCA: Principal component analysis
PCoA: Principal coordinates analysis
PE: Phosphatidylethanolamines
PG: Phosphatidylglycerol
PUFA: Long-chain polyunsaturated fatty acid
RMT: Rumen microbiota transplantation
RQUICKI: Quantitative insulin sensitivity check index
SAA: Serum amyloid A
SDL: Significantly different lipids
SOD: Superoxide dismutase
T-AOC: Total antioxidant capacity
TC: Total cholesterol
TG: Triglyceride
TMR: Total mixed ration
TNF-α: Tumor necrosis factor-α
TVFA: Total volatile fatty acid
VIP: Variable importance in the production
