# Supplementary material for: Repeated inoculation with rumen fluid accelerates the rumen bacterial transition with no benefit on production performance in postpartum Holstein dairy cows
Source: J Anim Sci Biotechnol. 2024 Feb 4;15:17. doi: 10.1186/s40104-023-00963-9 (PMC10838461; doi:10.1186/s40104-023-00963-9)
Supplement: Supplementary file 1 — Additional file 1: Table S1. Diet ingredients and chemical composition of TMR diet. [file 40104_2023_963_MOESM1_ESM.docx]

**Table S1** Diet ingredients and chemical composition of TMR diet

| **Item** | **% of dry matter basis** |
| --- | --- |
| Ingredients |  |
| Alfalfa hay | 11.72 |
| Oat hay | 2.73 |
| Corn silage | 26.04 |
| Steam-flaked corn | 18.45 |
| Yeast culture^1^ | 0.43 |
| Soybean meal | 17.80 |
| Cottonseed | 7.90 |
| Molasses | 2.69 |
| Ground corn | 3.91 |
| DDGS | 1.95 |
| Sprayed corn hull | 1.17 |
| 5% premix^2^ | 2.82 |
| Fat power^3^ | 1.74 |
| Sodium bicarbonate | 0.65 |
| Contents |  |
| Crude protein | 17.41 |
| Ether extract | 5.64 |
| Neutral detergent fiber | 28.54 |
| Acid detergent fiber | 19.89 |
| Net energy of lactation^4^, Mcal/kg | 1.82 |
| Calcium | 0.78 |
| Phosphorus | 0.43 |

^1^ Yeast culture XP product obtained from Diamond V Biological Fermentation Engineering Technology Co., LTD (Shenzhen, China)

^2^ The premix consists of mineral meal, calcium bicarbonate, sodium chloride, magnesium oxide, vitamin A, vitamin D3, vitamin E, calcium sulfate, magnesium sulfate, copper sulfate, zinc sulfate, manganese sulfate, sodium selenite, calcium phosphate, cobalt chloride, and yeast culture.

^3^ Fat power product obtained from Berg + Schmidt company (Germany)

^4^ Net energy of lactation was calculated based on values from NRC (2001) [21]
