# Supplementary figures and images for: Repeated inoculation with rumen fluid accelerates the rumen bacterial transition with no benefit on production performance in postpartum Holstein dairy cows
Source: J Anim Sci Biotechnol. 2024 Feb 4;15:17. doi: 10.1186/s40104-023-00963-9 (PMC10838461; doi:10.1186/s40104-023-00963-9)

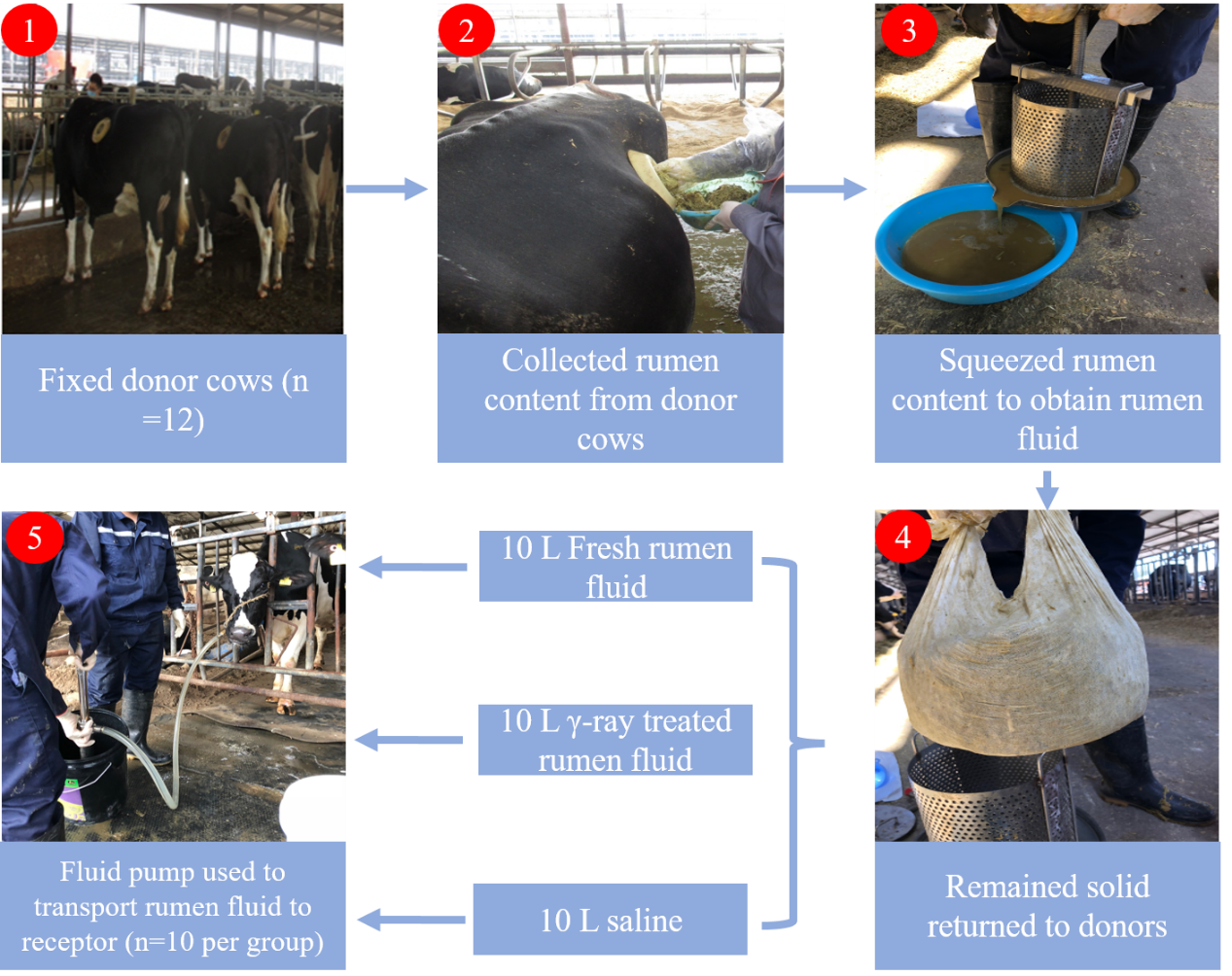


**Fig. S1** Rumen microbiota transplantation process

Supplement: Supplementary file 2 — Additional file 2: Fig. S1. Rumen microbiota transplantation process. [file 40104_2023_963_MOESM2_ESM.docx]

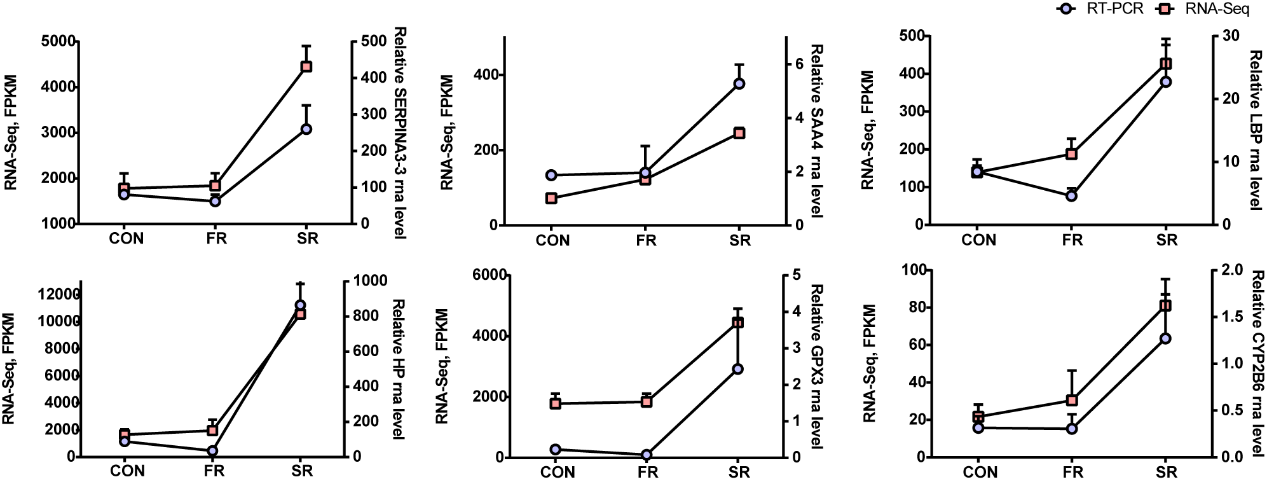
 **Fig. S2** Validation of RNA-Seq by RT-PCR

Supplement: Supplementary file 8 — Additional file 8: Fig. S2. Validation of RNA-Seq by RT-PCR. [file 40104_2023_963_MOESM8_ESM.docx]
