# Supplementary material for: Repeated inoculation with rumen fluid accelerates the rumen bacterial transition with no benefit on production performance in postpartum Holstein dairy cows
Source: J Anim Sci Biotechnol. 2024 Feb 4;15:17. doi: 10.1186/s40104-023-00963-9 (PMC10838461; doi:10.1186/s40104-023-00963-9)
Supplement: Supplementary file 3 — Additional file 3: Table S2. Primer sequences of validation genes. [file 40104_2023_963_MOESM3_ESM.docx]

**Table S2** Primer sequences of validation genes

| **Gene ID** | **Forward primer (5´→3´)** | **Reverse primer(5´→3´)** |
| --- | --- | --- |
| *GPx3* | TCTTCCGGGCATCCTGCCTT | TCAGTAGCTGGCCACGTTGA |
| *HP* | CCATACTGAATGAGAACACC | GAACCCAGTCCAGAATGGAG |
| *SAA4* | CGGTGTCTGGGCTGCAAAAA | TATTTCTTCGGCAGGCCCGC |
| *LBP* | AGTTGGACGATTCAATGTGG | TCAGGTACCGAACGTTGGTC |
| *CYP2B6* | ATGCTTTTATGCCCTTCTCC | GCAGGAACTGGATCCGATAG |
| *SERPINA3-3* | ACCTGGTAGTCTCCCAGGTG | CACTGGGGTTGGTGACTTTC |
| *Actin* | CATCCAGGAGCTGTTCAAGC | TCCTCACCGAAATCCTCTTC |
