# Supplementary material for: Repeated inoculation with rumen fluid accelerates the rumen bacterial transition with no benefit on production performance in postpartum Holstein dairy cows
Source: J Anim Sci Biotechnol. 2024 Feb 4;15:17. doi: 10.1186/s40104-023-00963-9 (PMC10838461; doi:10.1186/s40104-023-00963-9)
Supplement: Supplementary file 4 — Additional file 4: Table S3. Top 10 genera contributing to variance mostly among different groups. [file 40104_2023_963_MOESM4_ESM.docx]

**Table S3** Top 10 genera contributing to variance mostly among different groups

| **Taxonomy** | **CON 7 d vs. FR 7 d** | **CON 7 d vs. SR 7 d** | **FR 7 d vs. SR 7 d** | **CON 21 d vs. FR 21 d** | **CON 21 d vs. SR 21 d** | **FR 21 d vs. SR 21 d** | **Average** |
| --- | --- | --- | --- | --- | --- | --- | --- |
| *Succinivibrionaceae_UCG_001* | 20.10% | 19.95% | 12.45% | 31.56% | 29.62% | 28.10% | 23.63% |
| *Prevotella* | 15.56% | 16.23% | 16.10% | 11.69% | 10.32% | 11.64% | 13.59% |
| *Succinivibrionaceae_UCG_002* | 13.56% | 13.42% | 4.27% | 1.89% | 6.20% | 5.70% | 7.51% |
| *Rikenellaceae_RC9_gut_group* | 6.32% | 3.91% | 8.03% | 6.39% | 5.91% | 6.35% | 6.15% |
| *uncultured* | 5.21% | 5.43% | 2.11% | 1.22% | 1.41% | 1.13% | 2.75% |
| *Bacteroidales_RF16_group* | 4.74% | 6.26% | 8.22% | 4.18% | 4.72% | 4.36% | 5.41% |
| *F082* | 3.90% | 4.00% | 7.40% | 5.09% | 5.49% | 5.53% | 5.24% |
| *Prevotellaceae_Ga6A1_group* | 2.75% | 3.46% | 5.11% | 3.32% | 3.33% | 2.84% | 3.47% |
| *Treponema* | 2.34% | 1.24% | 3.17% | 4.71% | 4.36% | 3.80% | 3.27% |
| *Bacteroidales_BS11_gut_group* | 2.18% | 1.42% | 2.75% | 2.92% | 2.68% | 2.06% | 2.34% |
