# Supplementary material for: Repeated inoculation with rumen fluid accelerates the rumen bacterial transition with no benefit on production performance in postpartum Holstein dairy cows
Source: J Anim Sci Biotechnol. 2024 Feb 4;15:17. doi: 10.1186/s40104-023-00963-9 (PMC10838461; doi:10.1186/s40104-023-00963-9)
Supplement: Supplementary file 5 — Additional file 5: Table S4. Effects of fresh and sterile rumen microbiota transplantation on milk production and dry matter intake. [file 40104_2023_963_MOESM5_ESM.docx]

**Table S4** Effects of fresh and sterile rumen microbiota transplantation on milk production, dry matter intake and feed efficiency

| **Items** | **Groups** | | | **SEM** | ***P*-value** | | | | | |
| --- | --- | --- | --- | --- | --- | --- | --- | --- | --- | --- |
|  | **CON** | **FR** | **SR** |  | **Treatment** | **CON vs. FR** | **CON vs. SR** | | **Time** | **Treatment × Time** |
| Dry matter intake, kg/d | | |  |  |  |  |  |  | |  |
| Average | 13.26 | 13.11 | 14.27 | 0.385 | 0.07 | 0.96 | 0.16 | <0.01 | | 0.71 |
| d 1–3 | 8.51 | 7.86 | 10.00 | 0.527 | 0.98 |  |  |  | |  |
| d 4–6 | 11.08 | 11.58 | 12.02 | 0.554 | 1.00 |  |  |  | |  |
| d 7–9 | 11.91 | 12.72 | 13.52 | 0.574 | 1.00 |  |  |  | |  |
| d 10–12 | 14.04 | 13.79 | 13.67 | 0.571 | 1.00 |  |  |  | |  |
| d 13–15 | 15.36 | 13.24 | 17.02 | 0.586 | 0.48 |  |  |  | |  |
| d 16–18 | 14.83 | 15.96 | 17.23 | 0.605 | 0.99 |  |  |  | |  |
| d 19–21 | 17.07 | 16.65 | 16.45 | 0.613 | 1.00 |  |  |  | |  |
| Milk production, kg/d | | |  |  |  |  |  |  | |  |
| Average | 31.25^B^ | 28.86^C^ | 33.54^A^ | 0.572 | <0.01 | 0.02 | 0.04 | <0.01 | | 0.45 |
| d 1–3 | 15.69 | 14.22 | 20.62 | 0.951 | 0.38 |  |  |  | |  |
| d 4–6 | 27.37^AB^ | 25.09^B^ | 33.5^A^ | 0.962 | 0.04 |  |  |  | |  |
| d 7–9 | 29.70 | 29.01 | 34.18 | 0.922 | 0.72 |  |  |  | |  |
| d 10–12 | 32.85 | 30.52 | 34.26 | 0.933 | 0.99 |  |  |  | |  |
| d 13–15 | 37.05 | 31.81 | 35.50 | 0.945 | 0.76 |  |  |  | |  |
| d 16–18 | 37.60 | 33.99 | 39.02 | 0.988 | 0.90 |  |  |  | |  |
| d 19–21 | 38.49 | 37.41 | 37.69 | 1.011 | 1.00 |  |  |  | |  |
| Feed efficiency, milk production/dry matter intake | | | | |  |  |  |  | |  |
| Average | 2.54^AB^ | 2.30^B^ | 2.67^A^ | 0.093 | 0.02 | 0.16 | 0.65 | <0.01 | | 0.75 |
| d 1–3 | 2.04 | 1.60 | 2.40 | 0.139 | 0.68 |  |  |  | |  |
| d 4–6 | 2.88 | 2.39 | 3.17 | 0.149 | 0.85 |  |  |  | |  |
| d 7–9 | 2.75 | 2.45 | 2.94 | 0.145 | 0.99 |  |  |  | |  |
| d 10–12 | 2.42 | 2.54 | 2.78 | 0.144 | 1.00 |  |  |  | |  |
| d 13–15 | 2.60 | 2.52 | 2.33 | 0.148 | 1.00 |  |  |  | |  |
| d 16–18 | 2.75 | 2.29 | 2.43 | 0.150 | 0.99 |  |  |  | |  |
| d 19–21 | 2.34 | 2.31 | 2.63 | 0.157 | 1.00 |  |  |  | |  |

^A–C^Means within a row without a common letter differ (*P* < 0.05)
