# Supplementary material for: Repeated inoculation with rumen fluid accelerates the rumen bacterial transition with no benefit on production performance in postpartum Holstein dairy cows
Source: J Anim Sci Biotechnol. 2024 Feb 4;15:17. doi: 10.1186/s40104-023-00963-9 (PMC10838461; doi:10.1186/s40104-023-00963-9)
Supplement: Supplementary file 6 — Additional file 6: Table S5. Significantly different plasma lipids between the CON group and FR group. [file 40104_2023_963_MOESM6_ESM.docx]

**Table S5** Significantly different plasma lipids between CON group and FR group

| **Metabolites** | **FR mean** | **CON mean** | **VIP** | ***P*-value** | **Fold change** | **Model** |
| --- | --- | --- | --- | --- | --- | --- |
| LacCer(d16:2/26:1) | 0.00 | 0.01 | 1.99 | 0.05 | 0.52 | NEG |
| LPE(P-18:0/0:0) | 0.02 | 0.04 | 1.92 | 0.00 | 0.63 | POS |
| PC(P-22:0/17:2) | 0.97 | 1.52 | 2.42 | 0.00 | 0.64 | POS |
| CerP(d18:1/22:1) | 0.05 | 0.08 | 2.09 | 0.03 | 0.64 | POS |
| LPE(P-18:0/0:0) | 0.03 | 0.05 | 1.65 | 0.02 | 0.64 | NEG |
| SM(d14:1/26:0) | 0.06 | 0.10 | 1.58 | 0.02 | 0.65 | NEG |
| PC(P-20:0/18:4) | 1.53 | 2.36 | 2.21 | 0.00 | 0.65 | POS |
| SM(d15:2/27:0) | 2.13 | 3.29 | 1.98 | 0.02 | 0.65 | POS |
| LacCer(d14:2/22:0) | 0.01 | 0.01 | 1.59 | 0.02 | 0.66 | NEG |
| SM(d15:2/27:0) | 0.06 | 0.09 | 1.98 | 0.00 | 0.67 | NEG |
| SM(d15:1/27:0) | 0.65 | 0.98 | 2.08 | 0.01 | 0.67 | POS |
| GlcCer(d18:1/22:0) | 0.02 | 0.04 | 2.61 | 0.00 | 0.67 | POS |
| PC(26:1/13:0) | 0.12 | 0.18 | 2.13 | 0.01 | 0.67 | POS |
| PC(P-22:0/17:1) | 2.70 | 4.01 | 2.06 | 0.02 | 0.67 | POS |
| GlcCer(d18:1/24:0) | 0.02 | 0.04 | 1.87 | 0.02 | 0.68 | POS |
| PS(18:1/18:1) | 0.21 | 0.30 | 1.83 | 0.02 | 0.69 | POS |
| PC(O-22:2/16:1) | 0.27 | 0.39 | 1.80 | 0.02 | 0.69 | POS |
| PI(12:0/24:4) | 0.03 | 0.05 | 2.19 | 0.01 | 0.70 | POS |
| SM(d21:2/24:4) | 0.07 | 0.10 | 2.08 | 0.02 | 0.70 | POS |
| PE(20:4/20:4) | 0.03 | 0.04 | 1.25 | 0.03 | 0.71 | NEG |
| PE(O-22:2/18:2) | 0.03 | 0.04 | 1.57 | 0.02 | 0.71 | NEG |
| SM(d16:2/24:4) | 0.37 | 0.52 | 1.88 | 0.02 | 0.71 | POS |
| PC(P-18:0/20:4) | 0.35 | 0.49 | 1.84 | 0.03 | 0.72 | POS |
| PC(P-22:0/17:0) | 0.12 | 0.17 | 2.09 | 0.01 | 0.72 | POS |
| LPC(34:0/0:0) | 0.65 | 0.86 | 2.00 | 0.03 | 0.75 | POS |
| PC(30:0/2:0) | 1.53 | 2.02 | 2.06 | 0.01 | 0.75 | POS |
| PI(8:0/26:2) | 0.03 | 0.04 | 2.35 | 0.00 | 0.76 | POS |
| PC(3:0/36:0) | 0.06 | 0.08 | 1.84 | 0.05 | 0.76 | POS |
| PC(P-18:0/20:4) | 0.17 | 0.23 | 1.34 | 0.05 | 0.77 | NEG |
| SM(d16:2/26:1) | 0.45 | 0.58 | 1.67 | 0.01 | 0.77 | NEG |
| Cer(d18:1/16:0) | 0.02 | 0.03 | 1.92 | 0.04 | 0.78 | POS |
| PC(8:0/26:1) | 74.26 | 95.27 | 2.16 | 0.00 | 0.78 | POS |
| LacCer(d18:1/16:0) | 0.01 | 0.02 | 1.55 | 0.03 | 0.78 | POS |
| PC(P-22:0/11:0) | 5.73 | 7.29 | 1.76 | 0.03 | 0.79 | POS |
| SM(d18:2/26:1) | 0.03 | 0.04 | 1.76 | 0.03 | 0.80 | POS |
| SM(d22:1/18:2) | 0.04 | 0.05 | 1.42 | 0.03 | 0.82 | NEG |
| PC(18:2/16:0) | 32.97 | 37.38 | 1.40 | 0.05 | 0.88 | POS |
| PA(20:3/22:2) | 0.08 | 0.07 | 1.86 | 0.01 | 1.11 | NEG |
| PG(17:2/20:4) | 0.01 | 0.01 | 1.72 | 0.03 | 1.18 | NEG |
| SM(d14:0/17:0) | 0.01 | 0.01 | 1.64 | 0.02 | 1.18 | POS |
| PI(20:4/18:2) | 0.05 | 0.04 | 1.67 | 0.02 | 1.19 | NEG |
| PE(20:0/18:0) | 0.16 | 0.13 | 1.58 | 0.03 | 1.20 | NEG |
| PC(34:0/2:0) | 2.32 | 1.93 | 1.58 | 0.03 | 1.20 | NEG |
| SM(d14:0/18:2) | 0.07 | 0.06 | 1.64 | 0.03 | 1.22 | POS |
| PC(18:0/20:4) | 0.40 | 0.32 | 1.43 | 0.05 | 1.23 | POS |
| PE(P-20:0/18:1) | 0.03 | 0.02 | 1.79 | 0.02 | 1.23 | NEG |
| PE(26:1/10:0) | 1.19 | 0.96 | 1.38 | 0.03 | 1.23 | POS |
| LPC(17:0/0:0) | 0.32 | 0.26 | 1.38 | 0.03 | 1.24 | NEG |
| LPC(O-16:1/0:0) | 0.17 | 0.14 | 1.38 | 0.04 | 1.24 | NEG |
| SM(d14:1/20:1) | 3.42 | 2.73 | 1.67 | 0.02 | 1.25 | NEG |
| LPE(P-16:0/0:0) | 0.01 | 0.01 | 1.34 | 0.03 | 1.26 | NEG |
| LPC(20:0/0:0) | 0.06 | 0.04 | 2.15 | 0.01 | 1.26 | POS |
| PE(P-18:0/20:4) | 0.04 | 0.03 | 1.48 | 0.04 | 1.26 | NEG |
| PC(19:0/18:3) | 0.53 | 0.42 | 1.53 | 0.02 | 1.27 | POS |
| PC(P-20:0/22:6) | 0.02 | 0.01 | 1.61 | 0.05 | 1.27 | NEG |
| PC(P-22:0/9:0) | 0.06 | 0.04 | 1.63 | 0.02 | 1.27 | NEG |
| PI(18:0/18:1) | 0.07 | 0.06 | 1.47 | 0.05 | 1.28 | NEG |
| PI(14:0/24:4) | 0.04 | 0.03 | 1.67 | 0.04 | 1.31 | POS |
| PC(22:0/18:2) | 0.02 | 0.01 | 1.53 | 0.05 | 1.31 | NEG |
| PC(6:0/24:1) | 0.16 | 0.12 | 1.46 | 0.01 | 1.32 | POS |
| PC(19:0/20:4) | 0.04 | 0.03 | 1.54 | 0.04 | 1.32 | NEG |
| PC(O-18:2/14:1) | 0.02 | 0.01 | 1.67 | 0.02 | 1.32 | NEG |
| LPC(19:0/0:0) | 0.06 | 0.04 | 1.75 | 0.01 | 1.32 | POS |
| PG(18:3/18:0) | 0.02 | 0.01 | 1.62 | 0.05 | 1.33 | NEG |
| SM(d14:0/16:1) | 0.14 | 0.10 | 1.48 | 0.01 | 1.34 | POS |
| PC(18:0/18:4) | 2.14 | 1.59 | 1.75 | 0.03 | 1.34 | POS |
| LPE(18:1/0:0) | 0.04 | 0.03 | 1.92 | 0.01 | 1.35 | POS |
| PC(14:0/18:2) | 0.72 | 0.54 | 1.41 | 0.02 | 1.35 | POS |
| PI(12:0/26:2) | 0.25 | 0.19 | 1.69 | 0.01 | 1.35 | POS |
| PE(16:0/18:3) | 0.01 | 0.01 | 1.68 | 0.01 | 1.36 | NEG |
| PC(16:0/17:1) | 0.08 | 0.06 | 1.69 | 0.02 | 1.36 | NEG |
| PI(18:0/20:3) | 1.33 | 0.98 | 1.71 | 0.02 | 1.36 | POS |
| PE(22:5/22:5) | 0.04 | 0.03 | 1.85 | 0.01 | 1.36 | NEG |
| PC(P-18:0/2:0) | 0.06 | 0.04 | 1.81 | 0.01 | 1.36 | POS |
| PC(O-22:2/6:0) | 0.10 | 0.07 | 1.34 | 0.05 | 1.36 | POS |
| SM(d14:2/18:2) | 0.03 | 0.02 | 1.73 | 0.04 | 1.37 | NEG |
| SM(d14:1/18:2) | 0.02 | 0.01 | 1.52 | 0.05 | 1.37 | NEG |
| SM(d14:0/17:0) | 0.01 | 0.01 | 1.51 | 0.01 | 1.37 | NEG |
| SM(d14:0/17:1) | 0.06 | 0.04 | 1.61 | 0.01 | 1.38 | NEG |
| PC(15:0/20:4) | 0.01 | 0.01 | 1.75 | 0.01 | 1.38 | NEG |
| PC(22:6/18:1) | 0.01 | 0.01 | 1.93 | 0.02 | 1.38 | NEG |
| PI(16:0/15:0) | 0.01 | 0.01 | 1.76 | 0.01 | 1.39 | NEG |
| PC(27:0/2:0) | 0.04 | 0.03 | 1.61 | 0.02 | 1.39 | POS |
| PG(15:1/20:4) | 0.00 | 0.00 | 2.29 | 0.00 | 1.40 | NEG |
| PE(P-20:0/18:2) | 0.02 | 0.01 | 1.87 | 0.03 | 1.40 | NEG |
| PC(P-16:0/3:0) | 0.05 | 0.03 | 2.21 | 0.00 | 1.40 | NEG |
| SM(d17:0/18:2) | 0.08 | 0.06 | 1.39 | 0.04 | 1.40 | NEG |
| PC(O-16:2/20:5) | 0.03 | 0.02 | 1.74 | 0.01 | 1.40 | NEG |
| PI(18:0/22:5) | 0.24 | 0.17 | 1.78 | 0.03 | 1.40 | NEG |
| PC(22:5/14:0) | 0.73 | 0.52 | 1.41 | 0.04 | 1.42 | POS |
| PC(P-16:0/20:4) | 0.41 | 0.28 | 1.76 | 0.03 | 1.43 | POS |
| PC(P-16:0/3:0) | 0.06 | 0.04 | 1.81 | 0.02 | 1.43 | POS |
| LPC(20:1/0:0) | 0.02 | 0.02 | 1.82 | 0.02 | 1.43 | NEG |
| PC(16:0/20:4) | 1.77 | 1.23 | 1.99 | 0.04 | 1.44 | NEG |
| PC(18:3/22:5) | 2.57 | 1.79 | 1.60 | 0.03 | 1.44 | POS |
| PC(12:0/20:3) | 0.03 | 0.02 | 1.41 | 0.02 | 1.45 | POS |
| PE(20:2/19:0) | 0.02 | 0.02 | 1.83 | 0.04 | 1.45 | NEG |
| PE(17:1/16:0) | 0.01 | 0.01 | 1.41 | 0.05 | 1.46 | NEG |
| PG(17:0/18:0) | 0.04 | 0.03 | 1.73 | 0.01 | 1.47 | NEG |
| PC(16:0/16:1) | 0.03 | 0.02 | 1.85 | 0.00 | 1.47 | NEG |
| PE(17:1/18:1) | 0.04 | 0.02 | 2.08 | 0.00 | 1.48 | NEG |
| PC(O-22:2/6:0) | 0.03 | 0.02 | 1.79 | 0.01 | 1.48 | NEG |
| PC(26:2/6:0) | 0.19 | 0.13 | 1.71 | 0.00 | 1.48 | NEG |
| SM(d14:0/16:1) | 0.10 | 0.07 | 1.70 | 0.01 | 1.49 | NEG |
| PE(22:5/18:2) | 0.41 | 0.27 | 1.83 | 0.02 | 1.50 | NEG |
| PC(O-22:2/8:0) | 0.02 | 0.01 | 1.68 | 0.02 | 1.50 | NEG |
| PI(18:0/20:3) | 0.05 | 0.03 | 1.87 | 0.02 | 1.51 | NEG |
| SM(d15:2/18:2) | 0.01 | 0.01 | 1.68 | 0.03 | 1.51 | NEG |
| PE(15:0/18:2) | 0.08 | 0.06 | 1.39 | 0.05 | 1.52 | NEG |
| PE(20:5/18:1) | 0.04 | 0.02 | 1.78 | 0.00 | 1.52 | NEG |
| PC(14:1/16:0) | 0.05 | 0.03 | 2.03 | 0.00 | 1.53 | NEG |
| PC(20:0/18:2) | 1.33 | 0.87 | 1.85 | 0.01 | 1.53 | NEG |
| SM(d14:2/20:1) | 0.02 | 0.01 | 1.65 | 0.01 | 1.53 | NEG |
| PE(22:6/20:2) | 0.06 | 0.04 | 2.06 | 0.01 | 1.53 | NEG |
| SM(d14:2/19:0) | 0.04 | 0.03 | 1.72 | 0.03 | 1.53 | NEG |
| PE(20:2/20:0) | 0.09 | 0.06 | 1.87 | 0.02 | 1.54 | NEG |
| PG(18:1/20:4) | 0.02 | 0.02 | 1.51 | 0.04 | 1.55 | NEG |
| PC(2:0/17:2) | 0.11 | 0.07 | 1.81 | 0.04 | 1.55 | POS |
| PC(14:0/22:5) | 0.54 | 0.35 | 1.72 | 0.04 | 1.55 | POS |
| PI(18:0/20:2) | 1.23 | 0.79 | 1.78 | 0.01 | 1.56 | NEG |
| PI(17:0/16:0) | 0.04 | 0.02 | 1.79 | 0.01 | 1.56 | NEG |
| PC(13:0/18:1) | 0.22 | 0.14 | 1.81 | 0.01 | 1.57 | POS |
| PC(O-16:2/14:1) | 0.02 | 0.01 | 1.76 | 0.02 | 1.57 | NEG |
| PC(O-16:2/18:4) | 0.10 | 0.06 | 1.49 | 0.03 | 1.57 | NEG |
| PC(21:0/18:3) | 0.10 | 0.07 | 1.85 | 0.04 | 1.58 | POS |
| PC(22:5/18:2) | 0.02 | 0.01 | 2.04 | 0.01 | 1.59 | NEG |
| PC(18:0/20:5) | 1.33 | 0.84 | 2.17 | 0.00 | 1.60 | NEG |
| PC(16:1/20:5) | 0.02 | 0.01 | 2.21 | 0.00 | 1.60 | NEG |
| PI(18:2/20:0) | 0.09 | 0.06 | 1.81 | 0.01 | 1.61 | POS |
| LPE(20:3/0:0) | 0.08 | 0.05 | 1.46 | 0.05 | 1.62 | NEG |
| PC(O-18:2/18:4) | 0.05 | 0.03 | 1.75 | 0.02 | 1.62 | NEG |
| PE(20:1/19:0) | 0.02 | 0.01 | 1.53 | 0.03 | 1.63 | NEG |
| LPC(20:0/0:0) | 0.02 | 0.01 | 1.60 | 0.03 | 1.63 | NEG |
| LPC(16:1/0:0) | 0.98 | 0.60 | 2.44 | 0.00 | 1.64 | POS |
| LPE(18:2/0:0) | 1.09 | 0.66 | 1.83 | 0.01 | 1.64 | NEG |
| LPC(18:1/0:0) | 1.96 | 1.19 | 2.19 | 0.00 | 1.64 | POS |
| PE(18:0/20:5) | 0.04 | 0.02 | 2.35 | 0.00 | 1.65 | NEG |
| PC(12:0/26:2) | 13.27 | 8.04 | 1.59 | 0.04 | 1.65 | POS |
| PC(22:4/22:6) | 0.04 | 0.02 | 1.66 | 0.04 | 1.67 | NEG |
| PC(O-20:2/14:1) | 0.12 | 0.07 | 1.81 | 0.03 | 1.68 | NEG |
| PC(35:0/2:0) | 0.04 | 0.03 | 1.49 | 0.04 | 1.68 | NEG |
| LPC(22:6/0:0) | 0.01 | 0.01 | 2.01 | 0.02 | 1.69 | NEG |
| PC(16:0/20:5) | 0.83 | 0.49 | 2.25 | 0.00 | 1.70 | NEG |
| PC(P-16:0/2:0) | 17.02 | 9.99 | 2.43 | 0.00 | 1.70 | POS |
| PC(18:3/20:5) | 0.07 | 0.04 | 2.21 | 0.00 | 1.73 | POS |
| PI(18:0/20:1) | 0.04 | 0.02 | 2.12 | 0.01 | 1.76 | NEG |
| SM(d14:0/18:2) | 0.05 | 0.03 | 2.15 | 0.00 | 1.76 | NEG |
| PC(22:5/13:0) | 0.01 | 0.01 | 2.26 | 0.00 | 1.76 | NEG |
| LPE(15:0/0:0) | 0.02 | 0.01 | 2.18 | 0.00 | 1.76 | NEG |
| LPC(21:0/0:0) | 0.00 | 0.00 | 2.42 | 0.00 | 1.77 | NEG |
| SM(d17:0/24:4) | 0.16 | 0.09 | 1.65 | 0.04 | 1.77 | POS |
| PC(20:0/18:1) | 0.31 | 0.17 | 1.68 | 0.02 | 1.78 | NEG |
| PC(16:0/18:4) | 0.10 | 0.05 | 1.68 | 0.03 | 1.80 | NEG |
| LPC(17:1/0:0) | 0.10 | 0.06 | 2.05 | 0.00 | 1.80 | NEG |
| LPC(15:0/0:0) | 0.09 | 0.05 | 2.22 | 0.00 | 1.81 | POS |
| LPE(16:1/0:0) | 0.02 | 0.01 | 2.09 | 0.00 | 1.82 | NEG |
| PC(4:0/17:1) | 0.03 | 0.02 | 2.25 | 0.01 | 1.83 | POS |
| PE(20:1/20:0) | 0.03 | 0.01 | 1.88 | 0.01 | 1.84 | NEG |
| PC(O-16:2/2:0) | 27.90 | 15.15 | 2.24 | 0.00 | 1.84 | POS |
| LPE(20:4/0:0) | 0.31 | 0.17 | 2.12 | 0.00 | 1.86 | NEG |
| LPC(15:0/0:0) | 0.16 | 0.09 | 2.16 | 0.00 | 1.89 | NEG |
| LPC(18:1/0:0) | 6.13 | 3.23 | 2.56 | 0.00 | 1.90 | NEG |
| PE(P-18:0/20:3) | 0.08 | 0.04 | 1.73 | 0.02 | 1.91 | NEG |
| PC(13:0/22:5) | 0.05 | 0.03 | 2.18 | 0.04 | 1.93 | POS |
| LPC(22:4/0:0) | 0.05 | 0.03 | 1.85 | 0.02 | 1.95 | POS |
| PC(19:0/20:3) | 0.05 | 0.03 | 1.71 | 0.03 | 1.97 | NEG |
| PE(22:5/16:0) | 0.14 | 0.07 | 2.18 | 0.00 | 1.98 | NEG |
| PC(18:0/20:4) | 0.03 | 0.01 | 2.38 | 0.00 | 2.01 | NEG |
| LPC(14:0/0:0) | 0.27 | 0.13 | 2.57 | 0.00 | 2.02 | POS |
| LPC(20:4/0:0) | 0.51 | 0.25 | 2.05 | 0.00 | 2.02 | NEG |
| LPC(22:5/0:0) | 0.11 | 0.06 | 1.95 | 0.00 | 2.03 | NEG |
| PC(20:5/18:2) | 0.03 | 0.02 | 2.46 | 0.00 | 2.04 | NEG |
| LPE(20:5/0:0) | 0.02 | 0.01 | 2.22 | 0.00 | 2.06 | NEG |
| LPE(18:3/0:0) | 0.04 | 0.02 | 2.10 | 0.00 | 2.06 | NEG |
| PG(18:2/18:1) | 0.08 | 0.04 | 1.61 | 0.04 | 2.07 | NEG |
| PC(18:1/20:4) | 0.17 | 0.08 | 2.40 | 0.00 | 2.10 | NEG |
| PE(16:0/20:5) | 0.04 | 0.02 | 2.04 | 0.01 | 2.12 | NEG |
| PE(17:0/16:0) | 0.01 | 0.01 | 2.10 | 0.00 | 2.13 | NEG |
| LPC(20:4/0:0) | 0.17 | 0.08 | 2.33 | 0.00 | 2.15 | POS |
| LPC(14:0/0:0) | 0.03 | 0.02 | 2.34 | 0.00 | 2.19 | NEG |
| PC(18:3/18:2) | 0.20 | 0.09 | 2.37 | 0.00 | 2.25 | NEG |
| LPC(22:4/0:0) | 0.09 | 0.04 | 1.74 | 0.02 | 2.28 | NEG |
| LPC(17:2/0:0) | 0.01 | 0.00 | 2.47 | 0.00 | 2.28 | NEG |
| PC(21:0/18:2) | 0.01 | 0.00 | 1.88 | 0.03 | 2.28 | NEG |
| LPC(18:3/0:0) | 1.20 | 0.51 | 2.46 | 0.00 | 2.35 | POS |
| LPC(18:2/0:0) | 2.69 | 1.11 | 2.31 | 0.00 | 2.43 | POS |
| LPC(18:3/0:0) | 0.26 | 0.09 | 2.37 | 0.00 | 2.74 | NEG |
| PC(O-16:2/4:0) | 0.10 | 0.03 | 2.40 | 0.00 | 2.96 | POS |
| LPC(20:3/0:0) | 0.23 | 0.07 | 2.44 | 0.00 | 3.15 | POS |
| LPC(20:3/0:0) | 0.52 | 0.16 | 2.19 | 0.00 | 3.24 | NEG |
| LPC(20:2/0:0) | 0.05 | 0.01 | 2.07 | 0.03 | 3.36 | NEG |
