# Supplementary material for: Repeated inoculation with rumen fluid accelerates the rumen bacterial transition with no benefit on production performance in postpartum Holstein dairy cows
Source: J Anim Sci Biotechnol. 2024 Feb 4;15:17. doi: 10.1186/s40104-023-00963-9 (PMC10838461; doi:10.1186/s40104-023-00963-9)
Supplement: Supplementary file 7 — Additional file 7: Table S6. Significantly different plasma lipids between the CON group and SR group. [file 40104_2023_963_MOESM7_ESM.docx]

**Table S6** Significantly different plasma lipids between CON group and SR group

| **Metabolites** | **SR mean** | **CON mean** | **VIP** | ***P*-value** | **Fold change** | **Model** |
| --- | --- | --- | --- | --- | --- | --- |
| PC(O-16:2/26:2) | 0.01 | 0.01 | 1.87 | 0.03 | 0.61 | NEG |
| Cer(d18:0/23:0) | 0.10 | 0.14 | 1.56 | 0.04 | 0.72 | POS |
| SM(d14:1/26:0) | 0.07 | 0.10 | 1.57 | 0.05 | 0.73 | NEG |
| SM(d18:2/26:1) | 0.03 | 0.04 | 1.82 | 0.02 | 0.76 | POS |
| SM(d14:1/12:0) | 0.02 | 0.03 | 2.22 | 0.01 | 0.78 | POS |
| SM(d20:0/24:4) | 0.01 | 0.01 | 1.88 | 0.04 | 0.78 | NEG |
| DG(18:2/18:1/0:0) | 0.03 | 0.04 | 1.48 | 0.03 | 0.80 | POS |
| PC(30:0/2:0) | 1.64 | 2.02 | 1.75 | 0.02 | 0.81 | POS |
| Cer(d20:1/23:0) | 0.03 | 0.03 | 1.76 | 0.00 | 0.82 | POS |
| PC(37:0/2:0) | 0.06 | 0.08 | 1.26 | 0.04 | 0.83 | POS |
| PG(22:5/18:1) | 0.04 | 0.03 | 1.91 | 0.02 | 1.19 | NEG |
| PC(29:0/4:0) | 0.03 | 0.02 | 1.81 | 0.03 | 1.23 | NEG |
| SM(d14:1/26:0) | 0.28 | 0.22 | 2.24 | 0.04 | 1.29 | POS |
| LPC(18:1/0:0) | 4.25 | 3.23 | 1.70 | 0.02 | 1.32 | NEG |
| PC(21:0/18:2) | 0.01 | 0.00 | 1.39 | 0.05 | 1.33 | NEG |
| PC(17:2/2:0) | 0.03 | 0.02 | 1.32 | 0.03 | 1.39 | NEG |
| PC(O-22:2/6:0) | 0.03 | 0.02 | 1.23 | 0.04 | 1.44 | NEG |
| PE(18:0/14:0) | 0.02 | 0.01 | 1.34 | 0.03 | 1.47 | NEG |
| PC(O-16:2/18:2) | 0.23 | 0.15 | 2.94 | 0.00 | 1.48 | POS |
| SM(d14:1/14:0) | 0.05 | 0.04 | 1.30 | 0.03 | 1.49 | NEG |
| PC(10:0/20:3) | 0.04 | 0.03 | 2.10 | 0.04 | 1.53 | POS |
| PC(O-16:2/14:1) | 0.02 | 0.01 | 1.26 | 0.03 | 1.53 | NEG |
| PE(P-20:0/18:2) | 0.02 | 0.01 | 1.53 | 0.03 | 1.55 | NEG |
| PE(P-16:0/18:2) | 0.05 | 0.03 | 1.06 | 0.04 | 1.63 | NEG |
| PE(P-18:0/20:3) | 0.07 | 0.04 | 1.68 | 0.01 | 1.73 | NEG |
| PC(P-16:0/20:3) | 0.06 | 0.03 | 1.23 | 0.04 | 1.80 | NEG |
| PC(17:1/22:2) | 0.25 | 0.13 | 2.16 | 0.04 | 1.84 | POS |
